# Live and Heat-Treated *Lacticaseibacillus paracasei* NB23 Improve Selected Functional and Physiological Outcomes in Naturally Aged Mice

**DOI:** 10.3390/foods15203742

**Published:** 2026-10-20

**Authors:** Mon-Chien Lee, Ping-Jui Lin, Ting-Chun Lin, Chia-Hsuan Chou, Chien-Yu Hsiao, Chi-Chang Huang

**Affiliations:** 1 Graduate Institute of Sports Science, National Taiwan Sport University, Taoyuan City 333325, Taiwan; 2 Center for General Education, Taipei Medical University, Taipei 110301, Taiwan; 3 New Bellus Enterprise Co., Ltd., Tainan 720008, Taiwan; 4 Department of Nutrition and Health Sciences, Chang Gung University of Science and Technology, Kweishan, Taoyuan City 33303, Taiwan

**Keywords:** *Lacticaseibacillus paracasei* NB23, functional food ingredient, naturally aged mice, heat-treated bacteria, spatial learning, physical performance, exploratory microbiome associations

## Abstract

Live and heat-treated microbial preparations are candidate nutritional ingredients for supporting function during aging. This study evaluated *Lacticaseibacillus paracasei* NB23 in naturally aged male C57BL/6NCrl mice. Ten young mice served as a reference group, and forty aged mice received vehicle, live NB23 at 2.05 × 10^9^ or 6.15 × 10^9^ colony-forming units (CFU)/kg/day, or heat-treated NB23 at 6.15 × 10^9^ cell equivalents/kg/day for 12 weeks. Relative to aged controls, NB23-treated mice had shorter water-maze acquisition latency, longer treadmill endurance, higher hepatic antioxidant enzyme activities and glycogen, and lower selected whole-brain oxidative-damage markers. High-dose live and heat-treated NB23 also increased grip strength and muscle glycogen. Gastrocnemius myofiber area and selected femoral microarchitectural outcomes improved, whereas rotarod performance and tibialis anterior myofiber area did not. Exploratory analyses of brain and intestinal transcripts, fecal metabolites, and microbiota–host associations did not establish a gut-mediated mechanism. Among 325 taxon–outcome pairs in the exploratory microbiome–host screen, 49 met nominal *p* < 0.05, but none survived Benjamini–Hochberg correction (minimum adjusted *p* = 0.08967). Assay-specific subsets comprised four mice per group for micro-CT and five per group for qPCR, fecal metabolites, and microbiome sequencing, with two gene-specific qPCR exclusions. These findings support further evaluation of NB23 as a functional food ingredient, while the small, male-only animal study does not establish clinical anti-aging efficacy or equivalence of the live and heat-treated preparations.

## 1. Introduction

Biological aging is a progressive and multifactorial process characterized by the gradual loss of physiological integrity and a reduced capacity to maintain homeostasis under stress. Rather than affecting a single organ, aging involves interconnected alterations across the nervous, muscular, skeletal, metabolic, immune, and gastrointestinal systems. These changes arise from multiple interacting hallmarks, including genomic instability, loss of proteostasis, mitochondrial dysfunction, impaired nutrient sensing, cellular senescence, chronic inflammation, and gut microbial dysbiosis [1]. Persistent low-grade inflammation, commonly referred to as “inflammaging,” further contributes to tissue dysfunction, frailty, cardiovascular and metabolic disorders, and progressive loss of functional independence [2]. Therefore, interventions capable of preserving cognitive and physical function while supporting multiple organ systems may offer a more comprehensive strategy for promoting healthy aging than approaches directed toward a single molecular target.

The brain is particularly susceptible to age-related oxidative damage because of its high oxygen consumption, abundant oxidizable lipids, and comparatively limited regenerative capacity. Accumulation of reactive oxygen species can damage proteins, membrane lipids, and nucleic acids, leading to increased protein carbonylation, lipid peroxidation, and oxidative DNA lesions such as 8-hydroxy-2′-deoxyguanosine. These alterations can impair synaptic communication, mitochondrial function, and neuronal plasticity. Aging is also accompanied by deterioration of dopaminergic neurotransmission, and losses in dopamine receptors, transporters, and signaling capacity have been associated with declines in processing speed, working memory, executive function, and episodic memory [3]. Neuroplasticity-associated factors, including brain-derived neurotrophic factor, cAMP response element-binding protein, and synaptic proteins, as well as nuclear factor erythroid 2-related factor 2-regulated antioxidant responses, may therefore represent biologically relevant pathways linking oxidative homeostasis to cognitive resilience during aging.

Age-related cognitive decline frequently occurs together with impaired motor performance and reduced exercise capacity. Exercise-induced fatigue reflects a progressive reduction in the ability of the central nervous system and peripheral tissues to sustain a required level of force or power. Central fatigue may involve reduced neuronal excitability, altered motor cortical drive, and changes in neurotransmitter balance, whereas peripheral fatigue is influenced by energy-substrate depletion, metabolite accumulation, impaired excitation–contraction coupling, and muscle damage [4,5]. Because aging reduces neurological, metabolic, and muscular reserve, older individuals may be particularly vulnerable to these fatigue-related processes. Consequently, simultaneous assessment of spatial learning and memory, muscular strength, and endurance provides a broader evaluation of functional aging than any individual endpoint alone.

Skeletal muscle aging is characterized by progressive reductions in muscle mass, muscle-fiber size, strength, and contractile quality. These changes arise from the combined effects of motor-unit remodeling, impaired protein homeostasis, mitochondrial dysfunction, altered redox signaling, chronic inflammation, and diminished regenerative capacity [6]. Maintenance of hepatic and muscular glycogen stores is also important for sustaining energy availability during physical activity. An age-related decline in metabolic flexibility may compromise the ability to store and mobilize these energy reserves, thereby contributing to reduced endurance. At the structural level, muscle-fiber atrophy—particularly reductions in muscle cross-sectional area—provides histological evidence of declining muscle integrity. Accordingly, interventions that preserve antioxidant capacity, energy storage, muscle-fiber morphology, and functional performance may help counter several complementary features of age-related muscular decline.

Muscle deterioration is frequently accompanied by loss of bone mass and disruption of trabecular architecture. Aging alters the balance between bone formation and resorption, resulting in reductions in bone mineral density, increased trabecular separation, loss of trabecular connectivity, and greater susceptibility to skeletal fragility [7]. Skeletal muscle and bone are mechanically and metabolically coupled: loss of muscle strength reduces mechanical loading on bone, while deterioration of bone structure can further restrict mobility and physical activity. Evaluation of muscle and bone outcomes within the same experimental framework may therefore provide a more integrated representation of musculoskeletal aging.

In addition to these host-tissue changes, aging is associated with alterations in the intestinal environment. Studies in older adults have demonstrated that gut microbial composition is related to habitual diet, health status, systemic inflammation, and frailty, although these relationships are strongly influenced by residence, medication exposure, dietary intake, and other environmental factors [8,9]. Age-associated impairment of the intestinal barrier may permit greater translocation of microbial products and promote systemic inflammatory signaling. Conversely, microbial metabolites can communicate with distant organs through immune, neural, endocrine, and metabolic pathways. The microbiota–gut–brain axis includes signaling through the vagus nerve, immune mediators, neurotransmitter-related metabolites, and microbial fermentation products [10]. Similarly, the proposed gut–muscle axis may involve inflammation, nutrient availability, mitochondrial function, and anabolic signaling, although direct causal evidence in aging remains limited [11].

Short-chain fatty acids generated through microbial fermentation represent one possible link between the intestinal microbiota and host physiology. Acetate, propionate, butyrate, formate, and pentanoate can participate in intestinal epithelial metabolism, immune regulation, and host energy homeostasis. Propionate and butyrate have also been shown experimentally to inhibit osteoclast differentiation and bone resorption, suggesting that microbial metabolites may contribute to systemic regulation of bone remodeling [12]. Nevertheless, microbial composition and fecal metabolite concentrations do not necessarily reflect direct biological mediation of cognitive, muscular, or skeletal outcomes. Microbiota–host correlations should therefore be regarded as hypothesis-generating unless supported by multiplicity-adjusted statistical evidence and targeted mechanistic experiments.

Probiotic and postbiotic preparations have emerged as potential nutritional strategies for modifying host–microbial interactions and supporting healthy aging. Probiotics are live microorganisms that confer a health benefit when administered in adequate amounts, whereas the International Scientific Association for Probiotics and Prebiotics defines a postbiotic as a preparation of inanimate microorganisms and/or their components that confers a health benefit on the host [13]. Heat treatment eliminates microbial viability but may retain cell-wall structures, proteins, nucleic acids, and other biologically active components. For example, heat-killed *Lacticaseibacillus paracasei* D3-5 and its cell-wall lipoteichoic acid were reported to reduce intestinal permeability and inflammation and to improve physical and cognitive outcomes in aged mice [14]. However, biological activities are strain- and preparation-specific; findings obtained with one *L. paracasei* strain cannot be assumed to apply to another strain or to both live and heat-treated forms.

Several studies have demonstrated potentially relevant effects of *L. paracasei* PS23 in aging models. In senescence-accelerated mice, PS23 supplementation attenuated cognitive decline and was associated with changes in neural monoamines, antioxidant enzyme activities, and inflammatory mediators [15]. PS23 also attenuated age-related reductions in muscle mass and strength while preserving mitochondrial function and reducing oxidative and inflammatory stress [16]. A separate study reported that PS23 altered gut microbial composition and improved selected indices of gastrointestinal function in aged mice [17]. Both live and heat-killed PS23 preparations have additionally been investigated for their effects on recovery from exercise-induced muscle damage, supporting the possibility that microbial viability is not always required for physiological activity [18].

*L. paracasei* NB23 is a distinct strain isolated from a plant-based fermented product. In our previous study, supplementation with either live or heat-treated NB23 enhanced grip strength and endurance, increased hepatic and muscular glycogen storage, and attenuated exercise-associated fatigue biomarkers in mice without evidence of treatment-related toxicity [19]. More recently, a randomized controlled trial in healthy adults reported that NB23 supplementation was associated with improvements in lower-limb muscle strength and insulin-like growth factor 1-related signaling markers [20]. These findings support further investigation of NB23 as a nutritional intervention targeting physical and metabolic function. Nevertheless, whether live and heat-treated NB23 can attenuate the broader, multisystem consequences of biological aging has not been established.

Therefore, the present study investigated the effects of a 12-week intervention with low- and high-dose live NB23 or high-dose heat-treated NB23 in naturally aged male C57BL/6 mice. Young and naturally aged control groups were included to distinguish age-associated alterations from intervention-related responses. The principal outcomes encompassed spatial learning and memory, brain neurotransmitter and oxidative-damage indices, grip strength and treadmill endurance, hepatic antioxidant capacity, hepatic and muscular glycogen storage, skeletal-muscle histology, and femoral trabecular microarchitecture. Intestinal gene expression, fecal short-chain fatty acids, gut microbial composition, and microbiota–host correlations were evaluated as exploratory outcomes within an integrated analytical framework. We hypothesized that both live and heat-treated NB23 would attenuate selected age-related cognitive and physical impairments and improve brain, metabolic, muscular, and skeletal indices, while acknowledging that any observed associations involving the gut microbiota would not by themselves establish causal mediation.

## 2. Materials and Methods

### 2.1. Ethics Statement

All animal procedures were reviewed and approved by the Institutional Animal Care and Use Committee of National Taiwan Sport University, Taoyuan, Taiwan (approval no. IACUC-11318). The approved protocol was valid from 23 January to 31 December 2025. Animal care and experimental procedures were conducted in accordance with institutional guidelines and are reported following the ARRIVE 2.0 recommendations [21].

### 2.2. Preparation of Live and Heat-Treated Lacticaseibacillus paracasei NB23

*Lacticaseibacillus paracasei* NB23, originally isolated from a plant-based fermented product and identified as a Gram-positive lactic acid bacterium, was deposited in the Bioresource Collection and Research Center, Food Industry Research and Development Institute, Hsinchu, Taiwan, under accession number BCRC 911201 on 4 October 2023. NB23 was cultured anaerobically in Lactobacilli MRS broth (Difco Laboratories, Detroit, MI, USA) at 37 °C for 24 h until the stationary phase. The cells were collected by centrifugation at 3300× *g* for 10 min at 4 °C, washed twice with sterile saline, and resuspended. To prepare the heat-treated material, the NB23 cell suspension was heated at 70 °C for 1 h. This condition was selected based on in vitro splenocyte assays comparing the immunomodulatory effects of NB23 suspensions treated at different temperatures. Each preparation was then centrifuged to remove residual culture medium, mixed with a cryoprotectant, and freeze-dried. The resulting powders were dispensed into aliquots and stored at room temperature in a dry place protected from direct sunlight until use.

After freeze-drying, viable counts were determined by serial dilution and plating on MRS agar, followed by anaerobic incubation at 37 °C for 48 h. The viable count was 1 × 10^12^ CFU/g for the live NB23 powder and <1 × 10^3^ CFU/g for the heat-treated NB23 powder. This reported upper bound alone does not establish whether residual culturable cells were present. Total cell concentration was determined primarily by direct microscopic enumeration, with a Cellometer X2 fluorescent cell counter (Nexcelom Bioscience, Lawrence, MA, USA) using AO/PI staining as a complementary measurement. The heat-treated NB23 dose was calculated from the direct microscopic count and adjusted to 6.15 × 10^9^ cell equivalents/kg body weight/day. The preparation is therefore described as “heat-treated NB23,” without classifying it as completely inactivated.

New Bellus Enterprise Co., Ltd. (Tainan, Taiwan). supplied the intervention preparations under coded identities and retained the code key. The investigators receiving and administering the coded preparations were unaware of the identities of the live and heat-treated NB23 preparations during the animal experiment. Treatment identities were disclosed only after completion of the animal experiment. Thus, product-identity masking covered intervention administration and outcome acquisition during the experiment. The supplier was aware of the product-code correspondence. Product coding did not conceal the young-versus-aged status of the animals and is not described as masking the age-reference comparison.

### 2.3. Dose Conversion and Administration

The 1× human recommended intake was defined as 1 × 10^10^ CFU/day for a 60-kg adult. The corresponding mouse dose was calculated using the body-surface-area conversion factor of 12.3 [22]:

Mouse dose = (1 × 10^10^ CFU/day ÷ 60 kg) × 12.3 = 2.05 × 10^9^ CFU/kg body weight/day. Accordingly, the 1× live dose was 2.05 × 10^9^ CFU/kg BW/day, whereas the 3× live dose was 6.15 × 10^9^ CFU/kg BW/day. Heat-treated NB23 was administered at 6.15 × 10^9^ cell equivalents/kg BW/day. The 1× and 3× levels were selected to retain a human-intake-derived reference dose and a threefold higher exposure used in prior NB23 mouse work [19]. This scaling provides a translational starting point, not a pharmacokinetically validated or optimized dose range; no dose-ranging study was performed in aged mice.

Fresh dosing suspensions were prepared in the designated vehicle and administered once daily by oral gavage between 08:00 and 09:00 for 12 consecutive weeks. Individual doses were adjusted according to the most recently measured body weight, and the gavage volume did not exceed 10 mL/kg BW. Control mice received an equivalent volume of vehicle.

### 2.4. Animals, Housing, and Experimental Groups

Fifty male C57BL/6NCrl mice were obtained from the National Center for Biomodels, National Institutes of Applied Research, Taipei, Taiwan. Ten mice were 8 weeks old and served as the young reference group, whereas 40 naturally aged mice were 72 weeks old at the beginning of the intervention.

Animals were maintained in the animal facility of National Taiwan Sport University under controlled environmental conditions of 22 ± 2 °C, 60 ± 10% relative humidity, and a 12-h light/12-h dark cycle. Standard laboratory chow (LabDiet 5001 or equivalent formulation) and water were provided ad libitum.

The aged mice were allocated by body weight to four groups of ten mice each. The study records available for this revision do not document how the allocation sequence was generated or whether allocation was concealed. The five experimental groups were: YC: 8-week-old young control mice receiving vehicle; AC: 72-week-old naturally aged mice receiving vehicle; L1: aged mice receiving live NB23 at 2.05 × 10^9^ CFU/kg BW/day; L3: aged mice receiving live NB23 at 6.15 × 10^9^ CFU/kg BW/day; and HT3: aged mice receiving heat-treated NB23 at 6.15 × 10^9^ cell equivalents/kg BW/day. The young group was included as an age-reference group, whereas intervention contrasts compared each aged NB23 group with AC. For transparent presentation, spatial-learning acquisition, grip strength, rotarod performance, and treadmill endurance are treated as the primary functional-outcome family for this exploratory report; serum chemistry, tissue biochemistry, muscle morphology, and bone microarchitecture are treated as secondary supporting outcomes; and gene-expression, fecal-metabolite, microbiome, and host–microbiome analyses as exploratory outcomes. This reporting hierarchy is retrospective and does not imply that a single primary endpoint was prospectively specified or registered.

A literature-informed sample-size calculation was performed during manuscript revision using absolute forelimb grip strength as the reference functional outcome. Our separately published NB23 mouse study [19] reported group means of 134, 148, 150, and 158 g and corresponding SDs of 11, 13, 7, and 9 g (*n* = 10/group). The equal-weight grand mean is 147.5 g and the between-group mean variance is 74.75 g. To avoid relying on the smaller pooled SD, the largest published SD (13 g) was used as the common SD for a conservative external-data calculation, yielding Cohen’s f = √74.75/13 = 0.6651. This choice is grounded in the published variability; it does not assert that the variance in aged mice is known. Using the noncentral-F method implemented in G*Power 3.1 [23], a fixed-effects one-way omnibus ANOVA with four aged groups, α = 0.05, and target power = 0.90 required a minimum total *N* = 37. Equal allocation requires 40 mice, or ten per group, matching the actual aged cohort. At N = 40, modeled power is 0.9318 under these assumptions. The ten YC mice were an additional age-reference group and were not counted toward the treatment-group calculation. This calculation addresses the overall difference among the four aged groups.

The study cohort comprised 50 mice, with ten in each of YC, AC, L1, L3, and HT3. Core behavioral and serum-biochemistry datasets included ten mice per group; no animal-level exclusions were recorded for those analyses. Whole-brain biochemical, hepatic antioxidant, tissue-glycogen, and quantitative muscle-morphometry outcomes also included ten mice per group. To balance broad biological coverage with the processing requirements of complementary structural, molecular, and fecal analyses, assay-specific balanced subsets were used: four independent mice per group for femoral micro-CT and five independent mice per group for brain and intestinal qPCR, fecal short-chain fatty acids, and microbiome sequencing before qPCR quality filtering. Micro-CT involves individual high-resolution scanning, reconstruction, and region-of-interest analysis; molecular and fecal assays require separate specimen processing and analytical workflows. Using smaller balanced subsets for these exploratory endpoints allowed complementary tissue-level information to be obtained from the same animal cohort while retaining full-cohort assessment of the major functional outcomes.

### 2.5. Body Weight, Food Intake, and Water Intake

Body weight was measured before the intervention, weekly throughout the 12-week intervention, and at the end of the study. Food and water were supplied in measured amounts, and the remaining quantities were recorded daily. Daily intake was calculated by subtracting the remaining amount from the amount supplied and dividing by the number of mice and observation days.

Because food and water consumption was measured at the cage or group level rather than independently for each mouse, these intake measurements were summarized as group-level longitudinal observations and were not treated as independent mouse-level biological replicates.

### 2.6. Behavioral and Physical-Performance Assessments

Behavioral and physical-performance assessments were conducted during the final phase of the intervention. Apparatuses were cleaned between animals, and testing conditions were maintained as consistently as possible across groups.

#### 2.6.1. Morris Water-Maze Test

Spatial learning performance was evaluated using a reference-memory Morris water-maze task. A submerged escape platform remained in a fixed location in the third quadrant throughout the acquisition period. Each mouse underwent four trials per day for three consecutive days, with the four starting positions presented in randomized order.

For each trial, the mouse was released facing the pool wall and allowed up to 90 s to locate the hidden platform. Mice that located the platform were allowed to remain on it for 15–30 s. If a mouse failed to locate the platform within the allotted period, it was gently guided to the platform and allowed to remain there for the same period. An intertrial interval of approximately 30 min was used.

Mouse position and swimming trajectory were recorded using TopScanLite tracking software (version 2.0; CleverSys Inc., Reston, VA, USA). Escape latency was calculated for each trial and averaged within each day. Mean escape latency and mean path length across the three acquisition days were calculated at the mouse level. Swimming speed was not included as an outcome, and no probe trial was conducted.

#### 2.6.2. Forelimb Grip Strength

Forelimb grip strength was measured using a mouse grip-strength meter approximately 30 min after the designated daily administration. Each mouse was allowed to grasp the horizontal bar with both forepaws and was gently pulled backward by the tail until its grip was released. Peak force was recorded in grams.

Absolute grip strength was reported directly. Relative grip strength was calculated to account for differences in body mass:

Relative grip strength (%) = grip force (g) ÷ body weight (g) × 100.

#### 2.6.3. Accelerating Rotarod Test

Motor coordination and balance were evaluated using an accelerating rotarod apparatus (ROTA ROD; Panlab S.L.U., Barcelona, Spain). Mice were placed on the rotating rod at an initial speed of 4 rpm, which was gradually increased to 40 rpm over 300 s.

Each mouse completed three trials separated by 10-min rest periods. The latency to fall was recorded for each trial, and the average latency was used for analysis.

#### 2.6.4. Treadmill Running-to-Exhaustion Test

Whole-body endurance was assessed using a motorized multi-lane treadmill (MK680C; Muromachi Kikai Co., Ltd., Tokyo, Japan). Mice were fasted for 12 h before testing. The treadmill was set at a 15° incline, and the rear stimulus grid was operated at 2 Hz and 1.22 mA.

Running began at 10 m/min, after which speed was increased by 1 m/min every minute. Exhaustion was defined as remaining on the stimulus grid for more than 5 s without attempting to resume running despite stimulation. Time to exhaustion was recorded in minutes.

### 2.7. Terminal Sample Collection

Terminal sampling was performed approximately 30 min after the final administration. Mice were euthanized using carbon dioxide in accordance with the approved protocol. Approximately 0.6–0.8 mL of blood was collected by cardiac puncture into tubes without anticoagulant. Blood was allowed to clot for 30 min at room temperature and centrifuged at 4500 rpm for 15 min at 4 °C. The resulting serum was collected for biochemical analysis.

The liver, kidneys, heart, lungs, gastrocnemius muscle, tibialis anterior muscle, epididymal fat pad, brown adipose tissue, femora, intestinal tissue, fecal material, and whole brain were collected. Organs were rinsed with physiological saline, blotted dry, and weighed. Relative organ weight was calculated as:

Relative organ weight (%) = organ weight (g) ÷ terminal body weight (g) × 100.

Absolute and relative organ weights are reported in Table S1. Whole brains were collected without separation into the hippocampus, cortex, or other anatomical regions. Aliquots of the whole-brain samples were assigned to biochemical and gene-expression analyses and stored frozen until analysis.

### 2.8. Serum Biochemical Analyses

Serum biochemical measurements were performed using an automated clinical chemistry analyzer (Hitachi 717; Hitachi Ltd., Tokyo, Japan). The measured parameters included aspartate aminotransferase, alanine aminotransferase, blood urea nitrogen, creatinine, total protein, total cholesterol, triacylglycerol, high-density lipoprotein cholesterol, low-density lipoprotein cholesterol, and glucose.

### 2.9. Whole-Brain Dopamine and Oxidative-Damage Markers

Whole-brain biochemical analyses were conducted using ten mice per group. All brain samples were analyzed as whole-brain homogenates without regional dissection.

#### 2.9.1. Dopamine

Approximately 5–10 mg of whole-brain tissue was rinsed with ice-cold phosphate-buffered saline (PBS; 0.01 M, pH 7.4), minced, and homogenized in PBS on ice. Homogenates were further disrupted by sonication or freeze–thaw treatment and centrifuged at 5000× *g* for 5 min at 4 °C.

Dopamine concentrations in the supernatants were quantified using a commercial enzyme-linked immunosorbent assay kit (K4219; BioVision, Milpitas, CA, USA) according to the manufacturer’s instructions. Absorbance was measured at 450 nm. Whole-brain dopamine concentrations are expressed as ng/g tissue.

#### 2.9.2. Protein Carbonyls

Protein carbonyl content was measured using a colorimetric protein-carbonyl assay kit (no. 10005020; Cayman Chemical, Ann Arbor, MI, USA). Whole-brain tissue was homogenized in ice-cold potassium-phosphate buffer containing EDTA and phenylmethylsulfonyl fluoride and centrifuged at 10,000× *g* for 15 min at 4 °C.

Carbonyl groups were derivatized with 2,4-dinitrophenylhydrazine. Following protein precipitation and repeated washing with ethanol/ethyl acetate, the precipitate was dissolved in guanidine hydrochloride. Absorbance was measured within the manufacturer-recommended range of 360–385 nm. Results were normalized to protein concentration and expressed as nmol carbonyl/mg protein.

#### 2.9.3. Thiobarbituric Acid-Reactive Substances

Lipid peroxidation was assessed using a thiobarbituric acid-reactive substances assay kit (no. 10009055; Cayman Chemical). Whole-brain samples were homogenized under chilled conditions and centrifuged at 10,000× *g* for 10 min at 4 °C.

Malondialdehyde-containing samples and standards were reacted with thiobarbituric acid under acidic, high-temperature conditions. The resulting chromophore was quantified colorimetrically at 530–540 nm according to the manufacturer’s protocol. Values were normalized to tissue protein concentration.

#### 2.9.4. 8-Hydroxy-2′-Deoxyguanosine

Oxidized guanine immunoreactivity was measured using a competitive 8-hydroxy-2′-deoxyguanosine enzyme immunoassay kit (no. 589320; Cayman Chemical) according to the manufacturer’s instructions. Absorbance was measured at 405–420 nm, and concentrations were calculated from the standard curve and expressed in pg/mL. Because the antibody used in this assay may also recognize 8-hydroxyguanosine and 8-hydroxyguanine, the resulting measurements are interpreted as 8-OHdG-equivalent oxidized guanine immunoreactivity rather than as a mitochondria-specific measurement. No regional brain dissection or mitochondrial isolation was performed.

### 2.10. Hepatic Antioxidant Enzyme Activities

Liver tissue was homogenized under ice-cold conditions, and the supernatants were used to measure antioxidant enzyme activities with commercial kits from Cayman Chemical. Enzyme activities were normalized to tissue protein concentration and expressed as U/mg protein.

Superoxide dismutase activity was measured using kit no. 706002. The reaction was initiated with xanthine oxidase, incubated for 30 min at room temperature, and read at 440–460 nm.

Catalase activity was determined using kit no. 707002. Methanol and hydrogen peroxide were reacted with the tissue sample, and the formaldehyde product was detected using Purpald reagent. Absorbance was measured at 540 nm.

Glutathione peroxidase activity was measured using kit no. 703102 by monitoring the oxidation of NADPH in a coupled reaction at 340 nm.

Glutathione reductase activity was determined using kit no. 703202 by monitoring the rate of NADPH oxidation at 340 nm in the presence of oxidized glutathione.

### 2.11. Hepatic and Skeletal Muscle Glycogen

Liver and hindlimb skeletal muscle samples were collected approximately 30 min after the final administration, rinsed with saline, blotted dry, and stored at −80 °C until analysis. Tissue samples were homogenized in five volumes of extraction buffer using a Bullet Blender homogenizer (Next Advance Inc., Cambridge, MA, USA). Homogenates were centrifuged at 12,000× *g* for 15 min at 4 °C.

Glycogen concentrations in the resulting supernatants were determined using a chemical colorimetric assay. A calibration curve was prepared using purified glycogen standards (Sigma-Aldrich, St. Louis, MO, USA). Results were normalized to the amount of tissue analyzed and reported as tissue glycogen content.

### 2.12. RNA Extraction, Reverse Transcription, and Quantitative Real-Time PCR

#### 2.12.1. RNA Extraction and Reverse Transcription

RNA was extracted from 10–25 mg of intestinal mucosal tissue using an rSYNC RNA Isolation Kit (RSD300; Geneaid Biotech Ltd., New Taipei City, Taiwan). Tissue was homogenized in 400 μL of RS buffer containing 4 μL of dithiothreitol. Following the addition of 70% ethanol, the homogenate was transferred to a spin column. On-column DNase I digestion was performed for 15 min, and total RNA was washed and eluted in 50 μL of RNase-free water.

For complementary-DNA synthesis, 1 μg of total RNA was reverse-transcribed in a 20-μL reaction using the SensiFAST cDNA Synthesis Kit (BIO-65053; Meridian Bioscience, Cincinnati, OH, USA). Reverse transcription was performed at 25 °C for 10 min and 42 °C for 15 min, followed by enzyme inactivation at 85 °C for 5 min.

Whole-brain samples were processed without regional dissection. The same general RNA extraction, DNase treatment, and reverse-transcription workflow was used for the whole-brain gene-expression analyses.

#### 2.12.2. Quantitative Real-Time PCR

Quantitative real-time PCR was performed using a Rotor-Gene Q 2Plex instrument (Qiagen, Hilden, Germany). Each 10-μL reaction contained 2 μL of diluted cDNA, 1 μL each of 10-μM forward and reverse primers, 1 μL of nuclease-free water, and 5 μL of 2× Rotor-Gene SYBR Green PCR Master Mix (Qiagen; nos. 208054 and 981103). Samples were analyzed in technical duplicate.

Whole-brain expression of cAMP-responsive element-binding protein 1 (*Creb1*), brain-derived neurotrophic factor (*Bdnf*), synaptophysin (*Syp*), and nuclear factor erythroid 2-related factor 2 (*Nfe2l2*) was normalized to β-actin (*Actb*).

Intestinal expression of claudin-3 (*Cldn3*), claudin-5 (*Cldn5*), insulin-like growth factor 1 (*Igf1*), and peroxisome proliferator-activated receptor gamma coactivator 1-alpha (*Ppargc1a*) was normalized to *Actb*. Trefoil factor 3 (*Tff3*) expression was normalized to glyceraldehyde-3-phosphate dehydrogenase (*Gapdh*).

Technical-replicate Ct values were averaged within each mouse before calculating ΔCt (target minus reference). Relative expression was calculated as 2^−ΔΔCt^ using the mean AC ΔCt as calibrator [24]. In an exploratory post hoc reanalysis, a mouse–gene pair was excluded if either target or reference replicate was missing, nonnumeric, or had Ct ≥ 40; no other values were removed or imputed. This excluded AC mouse 4 for Cldn3 and L1 mouse 4 for Cldn5. All brain assays and other intestinal assays retained five mice per group. Figures display log2 relative expression to show the full range. Two-sided Welch tests on mouse-level ΔCt were followed by Benjamini–Hochberg correction separately for the four brain age contrasts, twelve brain treatment contrasts, five intestinal age contrasts, and fifteen intestinal treatment contrasts. These analysis families were specified for this reanalysis and are not described as prospectively registered. The initial biological sample size was five independent mice in each of YC, AC, L1, L3, and HT3; technical duplicates did not increase n. Following the stated rule, Cldn3 had n = 5, 4, 5, 5, and 5 and Cldn5 had n = 5, 5, 4, 5, and 5 in that group order. All other intestinal targets and all brain targets had n = 5 in each group.

### 2.13. Histological Examination and Muscle-Fiber Cross-Sectional Area

The liver, heart, kidneys, lungs, gastrocnemius muscle, tibialis anterior muscle, epididymal fat pad, and brown adipose tissue were fixed in 10% neutral-buffered formalin for at least 24 h. Samples were paraffin-embedded, sectioned, and stained with hematoxylin and eosin.

Liver, heart, kidney, lung, epididymal-fat-pad, and brown-adipose-tissue sections were examined for treatment-associated histopathological abnormalities and are presented in Figure S1.

For skeletal-muscle morphometry, transverse sections of the gastrocnemius and tibialis anterior muscles were imaged under identical acquisition settings. Myofiber boundaries were traced using ImageJ version 1.54f (National Institutes of Health, Bethesda, MD, USA). Cross-sectional area was calculated for individual fibers, and the animal-level mean was used as the experimental value. Quantitative CSA analyses included ten mice per group.

### 2.14. Femoral Micro-Computed Tomography

Excised femora were preserved in ethanol until analysis. One femur from each of four independent mice per group (20 mice in total) was evaluated using a SkyScan 1076 micro-computed-tomography system (Bruker, Kontich, Belgium). Scan settings were 50 kV tube voltage, 200 μA tube current, 0.5° rotation steps, 2.1-s exposure time, a 0.5-mm aluminum filter, and a nominal voxel size of 9 μm.

Images were reconstructed and analyzed using CTAn software (version 1.20.3.0; Bruker micro-CT, Kontich, Belgium). A consistent trabecular volume of interest in the distal femoral metaphysis was applied across all samples. Three-dimensional measurements included tissue volume, bone volume, bone volume fraction, bone surface, specific bone surface, trabecular thickness, trabecular separation, trabecular number, trabecular pattern factor, structure model index, and bone mineral density. Representative three-dimensional reconstructions were generated using identical visualization settings. Measurements and nomenclature followed established recommendations for rodent micro-CT analysis [25].

### 2.15. Fecal Short-Chain Fatty Acid Analysis

Fecal short-chain fatty acids were measured in five mice per group. Approximately 50 mg of fecal material was mixed with 10% aqueous isobutanol, homogenized, and centrifuged. A 675-μL aliquot of the supernatant was combined with 125 μL of 20-mM sodium hydroxide and 400 μL of chloroform. After vortex mixing and centrifugation, the upper aqueous phase was collected.

A 200-μL aliquot of the aqueous phase was mixed with 80 μL of isobutanol and 100 μL of pyridine, and the volume was adjusted to 650 μL with ultrapure water. Derivatization was initiated by adding 50 μL of isobutyl chloroformate. Samples were vortexed, sonicated for 1 min, and extracted with 150 μL of hexane. The upper hexane–isobutanol phase was collected for analysis.

Derivatized metabolites were analyzed using a Bruker gas chromatography–mass spectrometry system equipped with a VF-5ms capillary column. Peak integration was performed using Bruker MSWS version 8.2 software, and concentrations were calculated from the corresponding calibration data. Formate, propionate, butyrate, and pentanoate concentrations were reported in μM.

### 2.16. Full-Length 16S rRNA Gene Sequencing

Fecal microbiome analysis was performed in five mice per group. Total microbial DNA was extracted from fecal samples using a column-based fecal-DNA extraction method. DNA concentrations were measured using a Qubit 4 fluorometer (Thermo Fisher Scientific, Waltham, MA, USA) and adjusted to 1 ng/μL.

The full-length V1–V9 region of the bacterial 16S rRNA gene was amplified using barcoded primers based on the PacBio full-length 16S workflow. The primer structures were as follows:

- Forward: 5′-Phos/GCATC-[16-base barcode]-AGRGTTYGATYMTGGCTCAG-3′;
- Reverse: 5′-Phos/GCATC-[16-base barcode]-RGYTACCTTGTTACGACTT-3′.

Each PCR reaction contained 2 ng of genomic DNA and KAPA HiFi HotStart ReadyMix (Roche, Basel, Switzerland). Cycling conditions consisted of 95 °C for 3 min; 20–30 cycles of 95 °C for 30 s, 57 °C for 30 s, and 72 °C for 60 s; and a final extension at 72 °C for 5 min.

Amplicons were examined using 1% agarose-gel electrophoresis. Products with a predominant band at approximately 1500 bp were purified using AMPure PB beads. Equimolar barcoded amplicons were pooled, and 500–1000 ng of the pooled product was used for DNA-damage repair, end repair/A-tailing, and SMRTbell hairpin-adapter ligation.

After AMPure PB purification, the SMRTbell library was annealed using Sequel II Primer 3.1 and bound using Sequel II Binding Kit 3.1. Sequencing was performed in circular-consensus-sequencing mode using a PacBio Sequel IIe instrument to generate HiFi reads with a predicted Phred accuracy of at least Q30.

Demultiplexing, quality filtering, chimera removal, and species-level taxonomic assignment were performed as part of the sequencing bioinformatics workflow. Species-level relative-abundance data were used in the study’s exploratory host–microbiome correlation screen.

### 2.17. Host–Microbiome Correlation Analysis

The study’s exploratory correlation screen linked 13 bacterial species with 25 host outcomes using species-level relative-abundance values and the corresponding coefficient, nominal-*p*-value, and adjusted-*p*-value analysis matrices (BI-ANA-10766). Outcomes included physical performance, glycogen, water-maze measures, brain biomarkers, intestinal transcripts, muscle morphology, bone microarchitecture, fecal metabolites, and hepatic antioxidant enzyme activities.

The analysis matrices contained coefficients and nominal *p* values but not the individual matched observations needed to independently reconstruct the correlation estimator, covariate adjustment, missing-data handling, or pairwise sample sizes. We therefore characterize this as an exploratory, matrix-based correlation screen; it is not described as an adjusted or partial-correlation analysis.

We independently recalculated Benjamini–Hochberg adjusted *p* values across all 325 nominal comparisons; the results agreed with the adjusted values in the analysis matrix to numerical precision, and none was below 0.05. The heat map preserves the matrix order and uses a fixed correlation scale from −1 to +1; symbols indicate nominal *p* values only.

### 2.18. Statistical Analysis

Data are presented as mean ± standard deviation unless otherwise stated. The individual mouse was the experimental unit, except for food and water intake, which were recorded at the cage or group level. Across the cohort, 50 mice were included (YC, n = 10; AC, L1, L3, and HT3, n = 10 each). All animals contributed to the core behavioral and serum-biochemistry datasets (n = 10/group). Focused assay-specific subsets were used for micro-CT (n = 4/group), brain and intestinal qPCR (n = 5/group, except AC Cldn3 and L1 Cldn5, each n = 4 after the Ct-based quality rule in Section 2.12.2), and fecal SCFA and sequencing analyses (n = 5/group). These distinct specimen-specific endpoints broaden the physiological coverage of the study and are treated as exploratory tissue-level readouts, not separately powered mechanistic tests. No animal-level exclusions are recorded for the core analyses; the two documented qPCR mouse–gene exclusions are reported in Section 2.12.2. No missing values were imputed. The endpoint categories in Section 2.4 organize the current report and should not be interpreted as prospective endpoint registration. The sample-size justification and assay-subset rationale are provided in Section 2.4.

For the three-day Morris water-maze acquisition data, a two-factor mixed analysis of variance was used, with treatment group as the between-subject factor and training day as the within-subject factor. Mean path length across acquisition days was analyzed at the mouse level using Welch’s *t*-test for YC versus AC and Dunnett’s test for the three treatment-versus-AC comparisons.

For core functional outcomes, the age-associated contrast between YC and AC was assessed using a two-sided Welch’s *t*-test. The three intervention contrasts—L1 versus AC, L3 versus AC, and HT3 versus AC—were evaluated using Dunnett-adjusted comparisons. Mean differences and 95% simultaneous confidence intervals are reported for these functional contrasts where available.

Exploratory qPCR analyses used the reanalysis procedure in Section 2.12.2. The original analysis of the four fecal metabolite outcomes used Welch age contrasts and Dunnett treatment comparisons followed by Benjamini–Hochberg correction within endpoint families. Individual SCFA assay values and the complete statistical output were not included in the workbook supplied for this revision; numerical SCFA summaries are therefore reported descriptively. Micro-CT parameters were analyzed by one-way analysis of variance followed by the multiple-comparison procedure used to generate the original SAS (version 9.4; SAS Institute Inc., Cary, NC, USA) letter groupings; these analyses are considered exploratory because n = 4 per group.

For body weight, weekly values are presented descriptively. To avoid multiple independent tests across repeated weekly measurements, the individual change from baseline to final body weight was calculated and compared across groups using a one-way analysis of variance, with Welch’s *t*-test for YC versus AC and Dunnett-adjusted contrasts for L1, L3, and HT3 versus AC. This analysis summarizes net change and is not a model of the full longitudinal trajectory. Serum biochemical outcomes were analyzed using Welch’s *t*-test for YC versus AC and Dunnett’s test for the three treatment-versus-AC comparisons.

For the host–microbiome analysis, coefficients and nominal *p* values were taken from the available analysis matrices, and Benjamini–Hochberg adjusted *p* values were independently verified across 325 comparisons. Nominal associations are explicitly distinguished from FDR-significant results. Because no association survived correction and analytical details were unavailable in the aggregate matrices, these results are hypothesis-generating and cannot establish causal mediation. Other outcomes were analyzed as specified in the relevant figure and table legends. No experiment-wide multiplicity adjustment was applied across the full collection of physiological endpoints; this limitation is stated in the Discussion.

## 3. Results

### 3.1. Body Weight and Daily Food and Water Intake

Body-weight trajectories and daily food and water intake are summarized in Table 1. At baseline, the YC mice weighed 24.3 ± 1.5 g, whereas the aged groups had comparable mean body weights ranging from 35.8 to 36.3 g. Weekly body-weight values are presented descriptively. Mean change from baseline to final measurement was −0.33 ± 1.40 g in AC, 0.14 ± 1.19 g in L1, −0.19 ± 1.11 g in L3, and 0.40 ± 1.09 g in HT3. None of the treatment groups differed from AC in net change (Dunnett-adjusted *p* = 0.722, 0.987, and 0.404, respectively). The YC–AC difference in net change was significant (Welch *p* < 0.001). Final body weights were 35.7 ± 2.4, 36.4 ± 1.2, 36.0 ± 1.8, and 36.2 ± 1.6 g in AC, L1, L3, and HT3, respectively.

Group-level daily food and water intake appeared higher in YC than in the aged groups, whereas intake was similar among AC and the three NB23-treated groups. Because intake was recorded at the cage or group level rather than independently for each mouse, these results were interpreted descriptively. Net body-weight change likewise showed no evidence of a treatment-associated difference among aged mice; the weekly profile itself was not tested with a longitudinal mixed-effects model.

### 3.2. Serum Biochemistry, Organ and Tissue Weights, and Histological Assessment

Serum biochemical parameters are presented in Table 2. AST, total protein, triacylglycerol, and glucose did not show consistent treatment-associated changes among the aged groups. Serum ALT was modestly higher in AC than in YC, whereas none of the NB23-treated groups differed significantly from AC.

Serum BUN increased from 21.9 ± 2.7 mg/dL in YC to 28.7 ± 2.2 mg/dL in AC. Compared with AC, BUN was significantly lower in L3 and HT3, reaching 26.7 ± 1.8 and 25.2 ± 2.0 mg/dL, respectively, whereas L1 did not differ significantly from AC. Creatinine concentrations were comparable among AC and the three NB23-treated groups.

Total cholesterol was higher in AC than in YC but did not differ among AC, L1, L3, and HT3. LDL-C was also higher in AC than in YC; HDL-C and glucose did not differ significantly in the Welch age-reference comparisons, and none of these measures showed a consistent treatment-associated pattern among aged groups.

Absolute and body-weight-normalized organ and tissue weights are presented in Table S1. Compared with YC, the aged groups generally had greater absolute liver, kidney, and brown adipose tissue weights. Absolute gastrocnemius weight was lower in AC and L1 than in YC, whereas L3 and HT3 showed intermediate values. Relative gastrocnemius and brain weights were lower, and relative brown adipose tissue weight was higher, in all aged groups than in YC.

Most organ and tissue weights were comparable among AC, L1, L3, and HT3. Nevertheless, several isolated differences were identified. Absolute liver weight was lower in HT3 than in AC, whereas relative liver weight was lower in L3 and HT3 than in AC. Both absolute and relative lung weights were lower in HT3 than in AC and L3. Absolute epididymal fat-pad weight was higher in HT3 than in all other groups, and relative epididymal fat-pad weight was higher in HT3 than in AC and L3. Kidney, heart, gastrocnemius, tibialis anterior, brown adipose tissue, and brain weights otherwise showed no significant differences among the aged groups.

Representative H&E-stained sections of the liver, heart, kidneys, lungs, epididymal fat pad, and brown adipose tissue are shown in Figure S1. Visual examination did not reveal a consistent treatment-specific pattern of overt tissue disruption across the six tissues. Because quantitative or semiquantitative histopathological scoring was not performed, these representative images were used for descriptive assessment and were not interpreted as quantitative evidence of tissue protection. Overall, the isolated organ-weight differences were not accompanied by a consistent pattern of serum biochemical or histological abnormalities.

### 3.3. Live and Heat-Treated NB23 Improved Water-Maze Acquisition and Selected Physical Performance in Aged Mice

**Figure 1:**
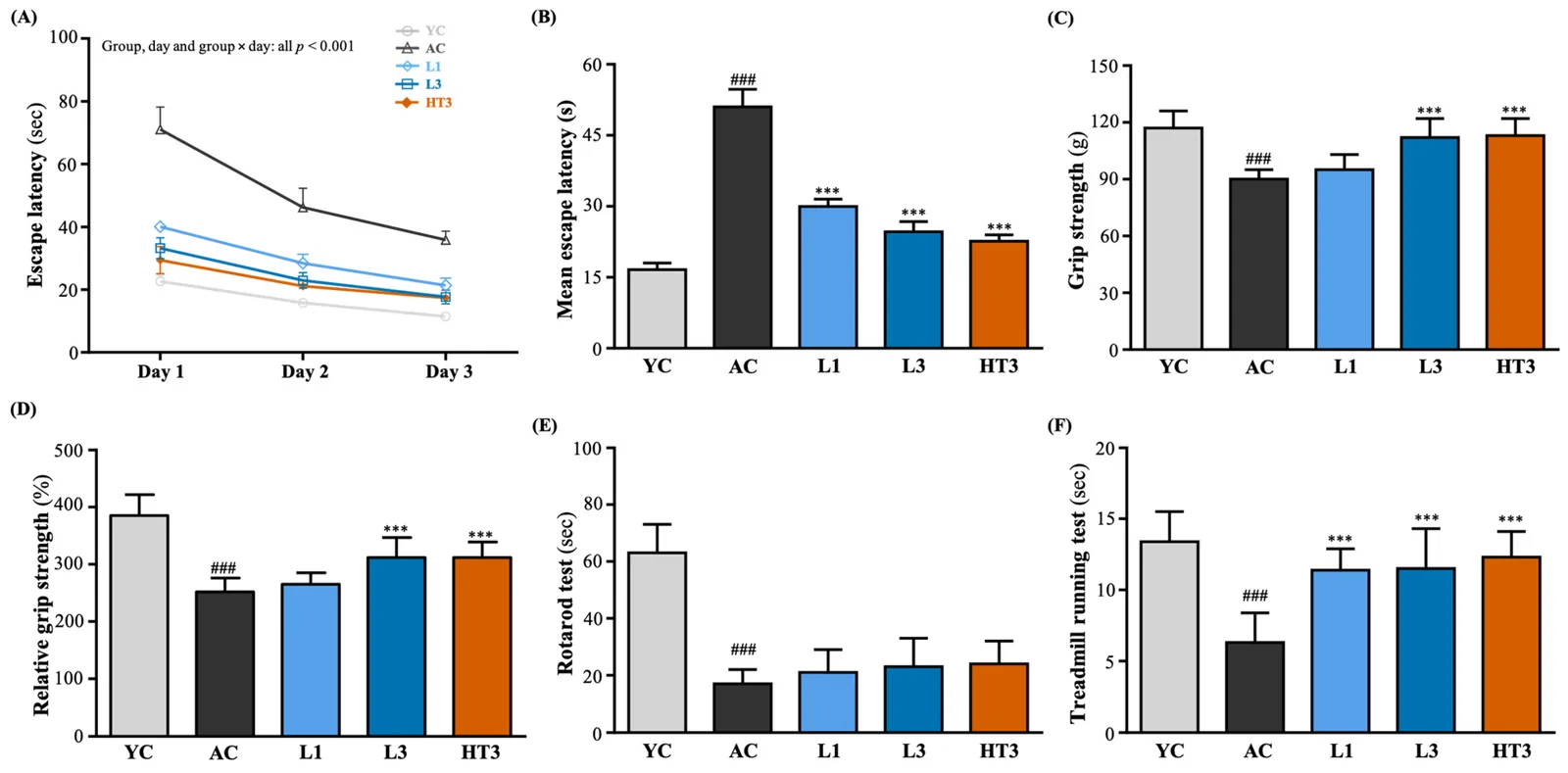
Effects of live and heat-treated *Lacticaseibacillus paracasei* NB23 on cognitive and physical performance in aged mice. (**A**) Escape latency during the three-day acquisition phase of the Morris water maze test. (**B**) Mean escape latency across the three acquisition days. (**C**) Absolute forelimb grip strength. (**D**) Relative grip strength normalized to body weight. (**E**) Latency to fall in the rotarod test. (**F**) Time to exhaustion in the treadmill test. Data are presented as the mean ± SD (n = 10 mice per group); individual points in panels (**B**–**F**) represent individual mice. Data in panel (**A**) were analyzed using a two-way mixed ANOVA, with group as the between-subject factor and day as the within-subject factor. Significant effects were detected for group, day, and the group × day interaction (all *p* < 0.001). For panels (**B**–**F**), AC was compared with YC using Welch’s *t*-test, whereas each NB23-treated group was compared with AC using Dunnett’s multiple-comparison test. ### indicate *p* < 0.001, respectively, versus YC; *** indicate *p* < 0.001, respectively, versus AC. YC, young control; AC, aged control; L1 and L3, live NB23 administered at the 1× and 3× doses, respectively; HT3, heat-treated NB23 administered at the 3× dose.

The effects of NB23 on spatial learning and physical performance are presented in Figure 1. During the three-day Morris water-maze acquisition period, significant effects were detected for group (F_4,45_ = 351.34, *p* < 0.0001), training day (F_2,90_ = 507.58, *p* < 0.0001), and the group × day interaction (F_8,90_ = 29.48, *p* < 0.0001; Figure 1A).

The mean escape latency across the three acquisition days was markedly longer in AC than in YC (51.01 ± 3.72 versus 16.64 ± 1.41 s, *p* < 0.0001). Compared with AC, mean escape latency was reduced by 41.3% in L1, 51.7% in L3, and 55.6% in HT3, with values of 29.95 ± 1.58, 24.62 ± 2.17, and 22.64 ± 1.33 s, respectively (all *p* < 0.0001; Figure 1B). For mean acquisition latency, Dunnett-adjusted mean differences versus AC were −21.06 s (95% simultaneous CI, −23.68 to −18.44) for L1, −26.40 s (−29.02 to −23.78) for L3, and −28.38 s (−31.00 to −25.76) for HT3 (all adjusted *p* < 0.0001). Mean path length across acquisition days showed the same direction of effect: 3.55 ± 0.29 m in AC, 2.24 ± 0.22 m in L1, 1.69 ± 0.21 m in L3, and 1.49 ± 0.17 m in HT3. Dunnett-adjusted mean differences versus AC were −1.31 m (95% simultaneous CI, −1.56 to −1.06), −1.86 m (−2.10 to −1.61), and −2.06 m (−2.31 to −1.81), respectively (all *p* < 0.0001).

Absolute forelimb grip strength was lower in AC than in YC (89.9 ± 5.3 versus 116.5 ± 8.8 g, *p* < 0.0001). L1 did not differ significantly from AC, whereas grip strength was 24.4% higher in L3 and 25.7% higher in HT3 than in AC (both *p* < 0.0001; Figure 1C). A similar pattern was observed after normalization to body weight. Relative grip strength was lower in AC than in YC, whereas L3 and HT3 exhibited approximately 24% higher relative grip strength than AC (both *p* < 0.0001; Figure 1D). The Dunnett-adjusted mean grip-strength differences versus AC were 5.0 g (95% simultaneous CI, −3.9 to 13.9) for L1, 21.9 g (13.0 to 30.8) for L3, and 23.1 g (14.2 to 32.0) for HT3. For treadmill time, the corresponding differences were 5.19 min (2.87 to 7.51), 5.24 min (2.92 to 7.56), and 6.00 min (3.68 to 8.32), respectively; all treatment-versus-AC comparisons had adjusted *p* < 0.001.

Rotarod latency was markedly shorter in AC than in YC (17.0 ± 4.6 versus 63.1 ± 10.2 s, *p* < 0.0001). Although slightly higher mean values were observed in the NB23-treated groups, none differed significantly from AC (Figure 1E).

Treadmill running time was also lower in AC than in YC (6.26 ± 2.05 versus 13.41 ± 2.08 min, *p* < 0.0001). Compared with AC, treadmill endurance increased by 82.8% in L1, 83.7% in L3, and 95.8% in HT3 (all *p* < 0.0001; Figure 1F). Thus, both live and heat-treated NB23 improved spatial-learning performance and endurance, whereas significant improvements in grip strength were primarily observed with the 3× live and heat-treated preparations.

### 3.4. NB23 Increased Whole-Brain Dopamine and Attenuated Selected Oxidative-Damage Markers

**Figure 2:**
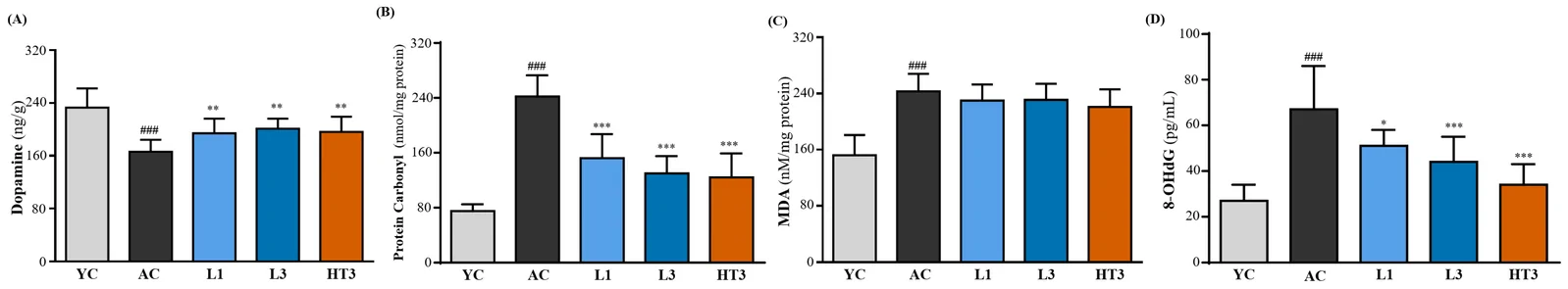
Effects of live and heat-treated NB23 on whole-brain (**A**) dopamine (ng/g), (**B**) protein carbonyls, (**C**) TBARS, and (**D**) 8-OHdG-equivalent oxidized guanine immunoreactivity. Brains were analyzed without regional dissection. The biochemical data and statistical symbols are retained from the original figures; dopamine units have been corrected. Brain qPCR is presented separately in Figure S3. Data are mean ± SD with individual mouse observations (n = 10/group). The original analysis compared YC with AC using a two-sided Welch test and each treatment with AC using Dunnett-adjusted contrasts. ### denote *p* < 0.001 for AC versus YC; *, **, and *** denote the corresponding thresholds for treatment versus AC.

Whole-brain dopamine and oxidative-damage markers are shown in Figure 2, and gene-expression outcomes are shown in Figure S3. Because the entire brain was homogenized without regional dissection, all findings represent whole-brain measurements.

Whole-brain dopamine was 28.9% lower in AC than in YC (165.9 ± 18.3 versus 233.3 ± 29.4 ng/g, *p* < 0.0001; Figure 2A). Compared with AC, dopamine concentrations were 16.7%, 20.9%, and 18.0% higher in L1, L3, and HT3, respectively (*p* = 0.0093, *p* = 0.0011, and *p* = 0.0051, respectively).

Protein carbonyl content was 3.21-fold higher in AC than in YC (242.0 ± 31.0 versus 75.3 ± 10.0 nmol/mg protein, *p* < 0.0001). Compared with AC, protein carbonyl content was reduced by 37.1% in L1, 46.4% in L3, and 48.7% in HT3 (all *p* < 0.0001; Figure 2B).

Whole-brain TBARS was 59.9% higher in AC than in YC (*p* < 0.0001). However, TBARS did not differ significantly between AC and L1, L3, or HT3 (*p* = 0.504, 0.535, and 0.108, respectively; Figure 2C).

Whole-brain 8-OHdG-equivalent immunoreactivity was 2.49-fold higher in AC than in YC (66.6 ± 19.0 versus 26.7 ± 7.0 pg/mL, *p* < 0.001). Compared with AC, 8-OHdG was reduced by 23.5% in L1, 34.6% in L3, and 49.6% in HT3 (*p* = 0.0230, *p* = 0.0007, and *p* < 0.0001, respectively; Figure 2D).

Whole-brain *Syp* expression was lower in AC than in YC after correction across the four age contrasts (*p* = 0.00364, adjusted *p* = 0.0146; Figure S3C). The *Creb1* age contrast was nominally significant (*p* = 0.0365) but did not survive correction (adjusted *p* = 0.0730; Figure S3A). *Bdnf* and *Nfe2l2* age contrasts were not significant (Figure S3B,D).

The L1 *Nfe2l2* contrast had nominal *p* = 0.0172 but adjusted *p* = 0.2064. Consequently, the neurochemical findings do not establish activation of the measured transcriptional pathways.

### 3.5. NB23 Enhanced Hepatic Antioxidant Enzyme Activities and Tissue Glycogen Stores

**Figure 3:**
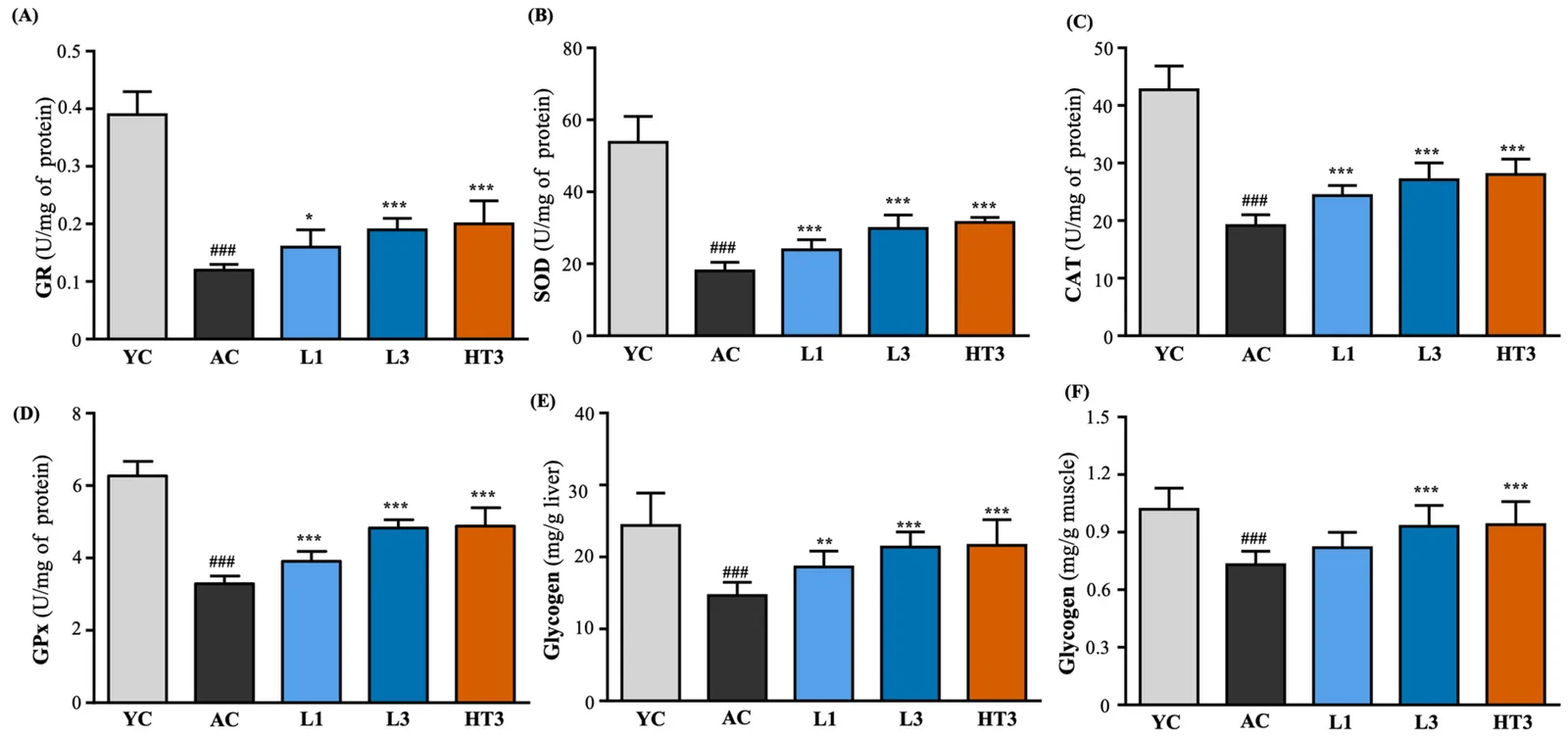
Effects of live and heat-treated *Lacticaseibacillus paracasei* NB23 on hepatic antioxidant enzyme activities and tissue glycogen contents in aged mice. Hepatic activities of (**A**) glutathione reductase (GR), (**B**) superoxide dismutase (SOD), (**C**) catalase (CAT), and (**D**) glutathione peroxidase (GPx). Glycogen contents in the (**E**) liver and (**F**) skeletal muscle were also determined. Data are presented as the mean ± SD (n = 10 mice per group), and individual points represent individual mice. AC was compared with YC using Welch’s *t*-test, whereas each NB23-treated group was compared with AC using Dunnett’s multiple-comparison test. ### indicate *p* < 0.001, respectively, versus YC; *, **, and *** indicate *p* < 0.05, *p* < 0.01, and *p* < 0.001, respectively, versus AC. YC, young control; AC, aged control; L1 and L3, live NB23 administered at the 1× and 3× doses, respectively; HT3, heat-treated NB23 administered at the 3× dose.

Hepatic antioxidant enzyme activities and tissue glycogen contents are presented in Figure 3. Aging was associated with marked reductions in glutathione reductase, superoxide dismutase, catalase, and glutathione peroxidase activities, with all four activities being significantly lower in AC than in YC (all *p* < 0.0001; Figure 3A–D).

Compared with AC, hepatic glutathione reductase activity was 32.7%, 52.7%, and 65.2% higher in L1, L3, and HT3, respectively (all *p* ≤ 0.0113; Figure 3A). Superoxide dismutase activity increased by 32.7%, 65.5%, and 74.8%, respectively (all *p* < 0.001; Figure 3B). Catalase activity increased by 27.2%, 41.5%, and 46.4% (all *p* < 0.0001; Figure 3C), whereas glutathione peroxidase activity increased by 18.9%, 47.1%, and 48.4%, respectively (all *p* < 0.001; Figure 3D). For each enzyme, the largest numerical improvement was observed in HT3.

Liver glycogen content was lower in AC than in YC (14.65 ± 1.83 versus 24.37 ± 4.50 mg/g liver, *p* < 0.0001). Compared with AC, liver glycogen increased by 26.9% in L1, 46.1% in L3, and 47.5% in HT3 (*p* = 0.0034, *p* < 0.0001, and *p* < 0.0001, respectively; Figure 3E).

Muscle glycogen was also lower in AC than in YC (0.73 ± 0.07 versus 1.02 ± 0.11 mg/g muscle, *p* < 0.0001). L1 did not differ significantly from AC. In contrast, muscle glycogen increased by 27.3% in L3 and 28.2% in HT3 (both *p* < 0.001; Figure 3F).

### 3.6. NB23 Preserved Gastrocnemius but Not Tibialis Anterior Myofiber Size

**Figure 4:**
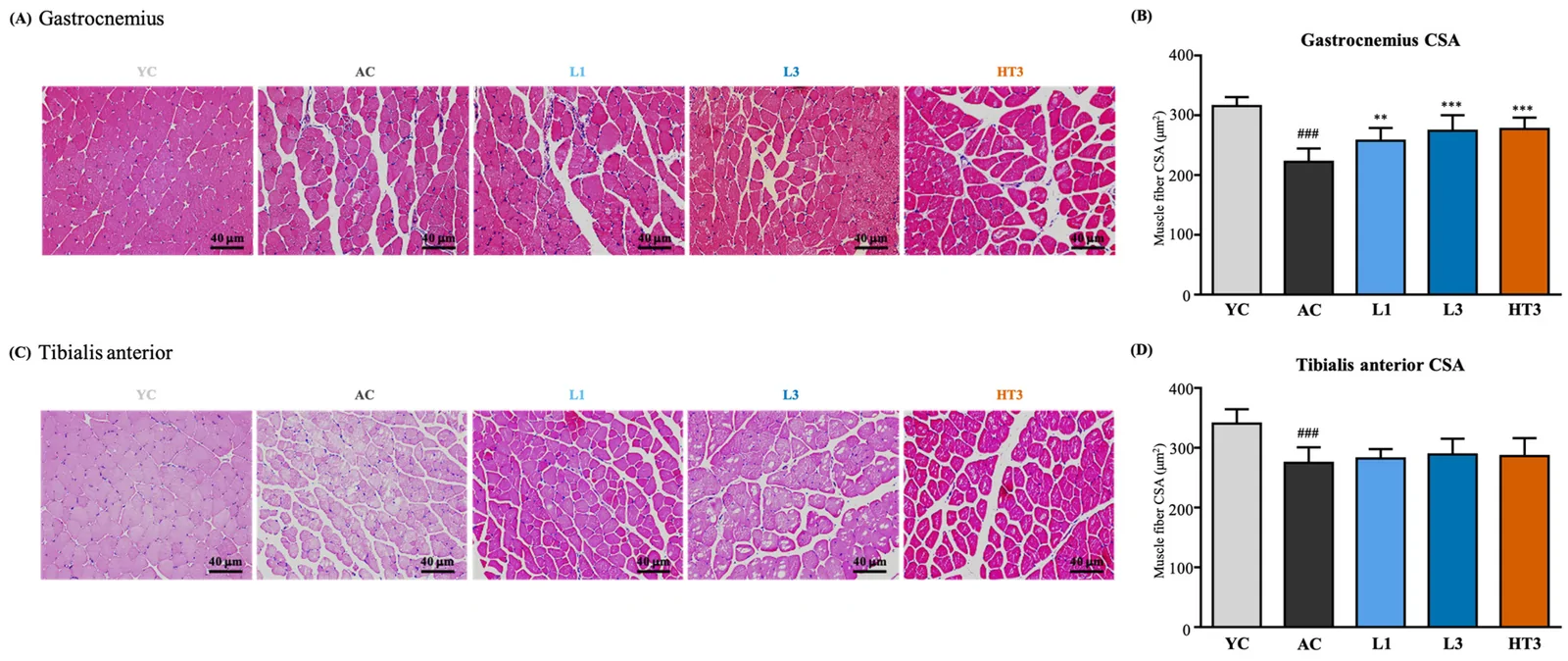
Effects of live and heat-treated *Lacticaseibacillus paracasei* NB23 on skeletal muscle fiber morphology and cross-sectional area in aged mice. Representative hematoxylin and eosin (H&E)-stained sections of the (**A**) gastrocnemius and (**C**) tibialis anterior muscles. Muscle fiber cross-sectional area (CSA) was quantified in the (**B**) gastrocnemius and (**D**) tibialis anterior muscles. Scale bars = 40 µm. Quantitative data are presented as the mean ± SD (n = 10 mice per group), and individual points represent individual mice. AC was compared with YC using Welch’s *t*-test, whereas each NB23-treated group was compared with AC using Dunnett’s multiple-comparison test. ### indicate *p* < 0.001, respectively, versus YC; **, and *** indicate *p* < 0.01, and *p* < 0.001, respectively, versus AC. YC, young control; AC, aged control; L1 and L3, live NB23 administered at the 1× and 3× doses, respectively; HT3, heat-treated NB23 administered at the 3× dose.

Representative H&E-stained sections and quantitative myofiber cross-sectional-area measurements are presented in Figure 4. Qualitatively, gastrocnemius sections from AC mice displayed smaller and more heterogeneous myofibers than those from YC mice. The NB23-treated groups, particularly L3 and HT3, showed a comparatively greater preservation of gastrocnemius myofiber size (Figure 4A,B).

Mean gastrocnemius CSA was 29.5% lower in AC than in YC (222.3 ± 22.3 versus 315.5 ± 15.1 μm^2^, *p* < 0.0001). Compared with AC, gastrocnemius CSA was 15.8% higher in L1, 23.3% higher in L3, and 24.7% higher in HT3 (*p* = 0.0034, *p* < 0.0001, and *p* < 0.0001, respectively; Figure 4B).

Tibialis anterior CSA was also lower in AC than in YC (274.7 ± 26.1 versus 340.4 ± 24.0 μm^2^, *p* < 0.0001). However, none of the three NB23-treated groups differed significantly from AC (Figure 4C,D). Thus, the observed myofiber-size response to NB23 was more evident in the gastrocnemius than in the tibialis anterior muscle.

### 3.7. NB23 Attenuated Age-Related Alterations in Femoral Trabecular Microarchitecture

**Figure 5:**
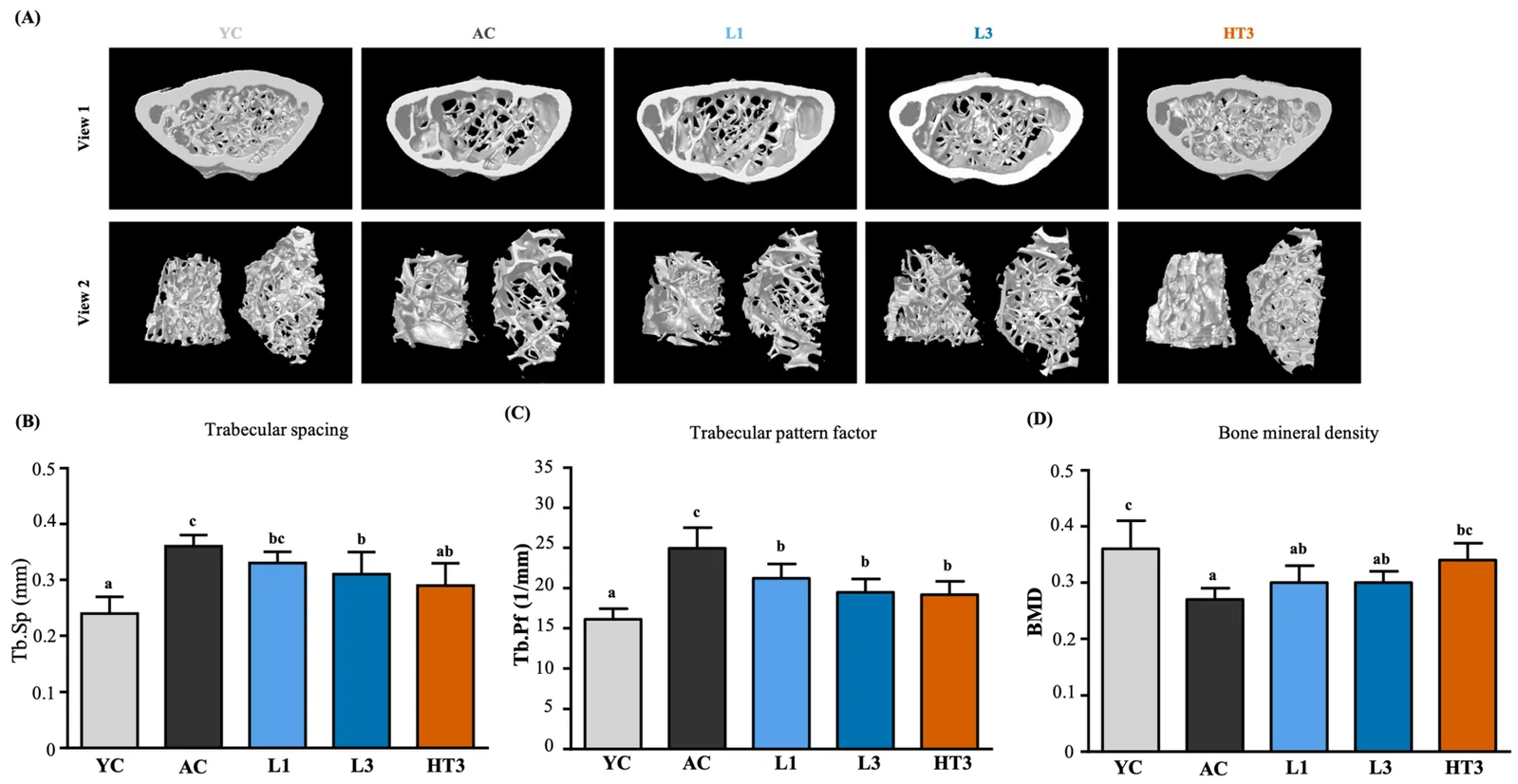
Effects of live and heat-treated *Lacticaseibacillus paracasei* NB23 supplementation on femoral bone microarchitecture in aged mice. (**A**) Representative three-dimensional femoral micro-CT reconstructions; two image views are shown for each experimental group. (**B**) Trabecular spacing (Tb.Sp). (**C**) Trabecular pattern factor (Tb.Pf). (**D**) Bone mineral density (BMD). Bars show mean ± SD, and open circles represent individual mice (n = 4/group). Different lowercase letters indicate significant differences among groups according to one-way ANOVA (*p* < 0.05). YC, young control; AC, aged control; L1, aged mice supplemented with live NB23 at the 1× dose; L3, aged mice supplemented with live NB23 at the 3× dose; HT3, aged mice supplemented with heat-treated NB23 at the 3× dose.

Femoral microarchitectural measurements are presented in Table 3, and representative three-dimensional reconstructions and selected outcomes are shown in Figure 5. Compared with YC, AC exhibited lower bone volume, bone-volume fraction, bone surface, trabecular number, and bone mineral density, together with higher trabecular separation and trabecular pattern factor. Tissue volume, specific bone surface, trabecular thickness, and structure model index did not differ significantly among groups.

Trabecular separation increased from 0.239 ± 0.031 mm in YC to 0.357 ± 0.019 mm in AC. Compared with AC, trabecular separation was 13.6% lower in L3 and 18.9% lower in HT3, whereas L1 did not differ significantly from AC (Figure 5B).

Trabecular pattern factor increased from 16.11 ± 1.33 mm^−1^ in YC to 24.96 ± 2.55 mm^−1^ in AC. Compared with AC, it was reduced by 14.9% in L1, 22.0% in L3, and 23.1% in HT3 (Figure 5C).

Bone mineral density was lower in AC than in YC. L1 and L3 exhibited intermediate mean values but did not differ significantly from AC. In contrast, BMD was 24.0% higher in HT3 than in AC and did not differ significantly from YC (Figure 5D). These micro-CT findings indicate that the higher live dose and heat-treated preparation were associated with greater preservation of selected trabecular outcomes, with the most consistent BMD response observed in HT3. These findings should nevertheless be interpreted in the context of the micro-CT sample size of four mice per group.

### 3.8. Intestinal Gene Expression and Fecal Short-Chain Fatty Acids

**Figure 6:**
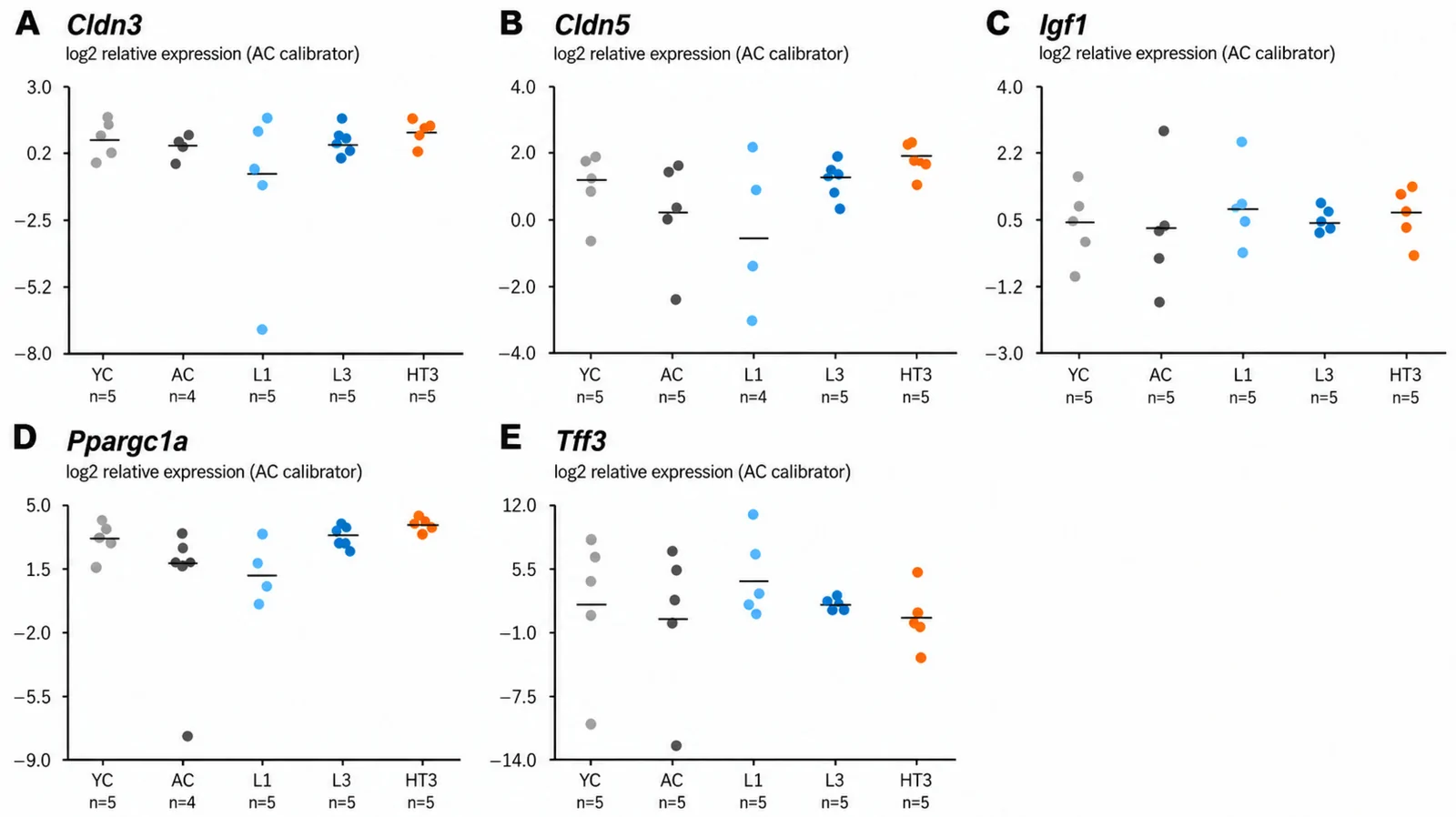
Exploratory intestinal qPCR reanalysis. (**A**) Cldn3, (**B**) Cldn5, (**C**) Igf1, (**D**) Ppargc1a, and (**E**) Tff3. Points show individual mice on a log2 relative-expression scale, with mean AC ΔCt as calibrator; horizontal lines show the mean log2 expression. Actb was the reference gene for panels (**A**–**D**) and Gapdh for panel (**E**). Sample sizes are shown below each group (five/group except AC Cldn3 and L1 Cldn5, four/group). The Ct ≥ 40 exclusion rule and duplicate averaging are described in Section 2.12.2. Two-sided Welch tests on ΔCt were followed by Benjamini–Hochberg correction separately across five age and fifteen treatment contrasts. No contrast met adjusted *p* < 0.05. Fecal metabolite panels are in Figure S2.

Exploratory reanalysis of intestinal Ct data is presented in Figure 6. No age contrast or NB23 treatment contrast for Cldn3, Cldn5, Igf1, Ppargc1a, or Tff3 survived correction within its respective comparison family.

The HT3–AC Cldn3 contrast had nominal *p* = 0.0266 but adjusted *p* = 0.3994. The corresponding Cldn5 contrast had *p* = 0.0783 and adjusted *p* = 0.5764. Ppargc1a and Tff3 showed particularly wide mouse-level variation. These data do not establish improved intestinal permeability or mitochondrial function. Technical-replicate discrepancies and the post hoc exclusion rule limit interpretation.

Fecal formate, propionate, butyrate, and pentanoate concentrations also showed considerable interindividual variation (Figure S2). HT3 exhibited a numerically higher formate concentration than AC (50.1 ± 15.6 versus 24.5 ± 5.1 μM), but this comparison was not significant after correction (nominal *p* = 0.0526, *q* = 0.631). No significant treatment-associated differences were observed for propionate, butyrate, or pentanoate.

### 3.9. Exploratory Microbiota–Host Associations

**Figure 7:**
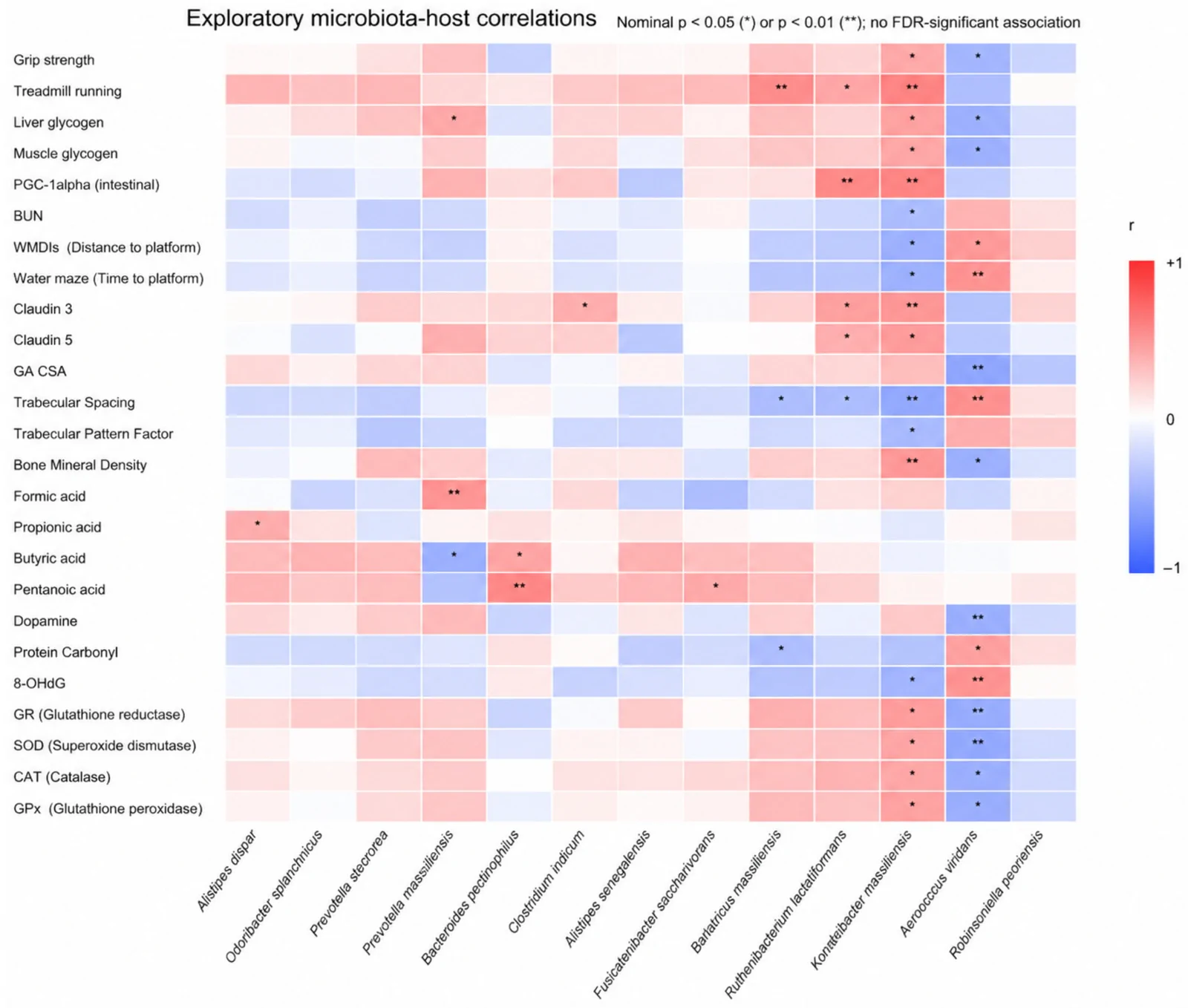
Exploratory taxon–outcome correlation screen. The heat map summarizes coefficients for 13 bacterial species and 25 host outcomes (325 pairs); red and blue indicate positive and negative coefficients on a fixed scale from −1 to +1. Asterisks denote nominal *p* < 0.05 and *p* < 0.01. No pair survived Benjamini–Hochberg correction (minimum adjusted *p* = 0.08967). The matrix-level output does not contain individual matched observations, so per-pair n and covariate adjustment could not be reconstructed. The display is hypothesis-generating and does not imply mechanistic links. PGC-1α and Claudin-3/5 refer to intestinal transcript measures.

The exploratory taxon–outcome screen covered 325 pairs linking 13 bacterial species with 25 host outcomes (Figure 7). Forty-nine pairs had nominal *p* < 0.05, but none survived Benjamini–Hochberg correction (minimum adjusted *p* = 0.08967). The screen is presented to identify candidates for replication; it does not show that individual taxa mediate NB23-associated physiological outcomes.

## 4. Discussion

This study evaluated whether 12 weeks of supplementation with live or heat-treated *L. paracasei* NB23 could attenuate functional and tissue-level features of aging in naturally aged male C57BL/6NCrl mice. The principal finding was a coordinated, although incomplete, preservation of several aging-sensitive outcomes. Compared with aged controls, NB23-treated mice showed shorter escape latencies during Morris water maze acquisition, longer treadmill endurance, greater hepatic antioxidant enzyme activities, higher hepatic glycogen stores, lower whole-brain protein and DNA oxidation, and partial preservation of femoral trabecular microarchitecture. Improvements in grip strength, muscle glycogen, and several other outcomes were most consistent in the high-dose live (L3) and heat-treated (HT3) groups. Conversely, rotarod performance, tibialis anterior myofiber cross-sectional area, whole-brain TBARS, the exploratory brain and intestinal qPCR panels, fecal short-chain fatty acids (SCFAs) did not provide consistent treatment evidence; microbiota–host correlations also failed FDR correction. Accordingly, the present findings support a broad functional-preservation phenotype rather than uniform reversal of biological aging, and they identify several mechanistic hypotheses that require direct testing.

The physical-performance findings are consistent with previous strain-specific studies of lactobacilli in aging and exercise models. Live *L. paracasei* PS23 attenuated muscle loss and weakness in SAMP8 mice in association with improved mitochondrial function and reduced inflammatory and oxidative stress [16], whereas *Lactiplantibacillus plantarum* TWK10 improved muscle strength, bone phenotypes, and cognitive performance in aged mice [26]. The same NB23 strain used here has also been reported to enhance endurance and reduce exercise-associated fatigue markers in younger mice [19]. In the current naturally aged model, all three NB23 preparations prolonged treadmill time to exhaustion, but absolute and body-weight-normalized grip strength increased only in L3 and HT3, and rotarod performance was not significantly improved. This divergence suggests that endurance, force production, and motor coordination were not affected identically. Morphologically, gastrocnemius myofiber cross-sectional area increased in all NB23 groups, whereas the tibialis anterior endpoint did not differ significantly among aged groups, indicating that the structural response was muscle-specific rather than generalized. The absence of treatment-related differences in body weight, food intake, or water intake among aged groups further argues against greater caloric intake or weight loss as the primary explanation for the functional findings.

Energy availability may have contributed to the endurance phenotype. Hepatic glycogen was higher in all NB23 groups than in aged controls, whereas muscle glycogen increased significantly in L3 and HT3, paralleling the stronger grip-strength response at these exposures. Glycogen is a major substrate for sustained exercise, and previous work with TWK10 demonstrated that lactobacillus supplementation can improve exercise performance together with energy storage and utilization [27]. Nevertheless, the present study did not quantify substrate oxidation, mitochondrial respiration, insulin signaling, or glycogen turnover; therefore, the glycogen data should be interpreted as a plausible physiological correlate rather than proof of a causal pathway. Experimental work in germ-free and colonized mice supports a role for the gut microbiota in regulating skeletal muscle mass, mitochondrial function, and energy metabolism [28]. However, because the intestinal gene-expression, fecal SCFA, and FDR-adjusted microbiome-correlation analyses in this study were not significant after multiplicity correction, a microbiota-mediated gut–muscle mechanism cannot be concluded from the current dataset.

NB23 supplementation also improved several behavioral and biochemical outcomes related to brain aging. All treated groups displayed shorter escape latencies across Morris water maze acquisition, accompanied by higher whole-brain dopamine and lower protein carbonyl and 8-hydroxy-2′-deoxyguanosine concentrations than aged controls. These findings are directionally consistent with the reported attenuation of age-related cognitive decline, oxidation, and inflammation by *L. paracasei* PS23 in SAMP8 mice [15]. However, the response was selective: whole-brain TBARS was not significantly reduced by NB23, and treatment-related changes in *Creb1*, *Bdnf*, *Syp*, and *Nfe2l2* mRNA did not remain significant after Benjamini–Hochberg adjustment. Thus, the data support changes in whole-brain neurochemical and oxidative-damage measures, but they do not establish activation of a CREB–BDNF synaptic-plasticity program or an NRF2 transcriptional mechanism. Moreover, because whole brains were homogenized without regional dissection, localized responses in the hippocampus, striatum, or cortex may have been diluted or obscured. Vagal signaling and microbiota-derived metabolites are biologically credible routes through which intestinal microbes can influence the central nervous system [29,30], but these routes remain hypotheses in the present study, particularly because the measured fecal SCFAs were unchanged after correction for multiple testing.

The hepatic antioxidant data provide an additional systemic context for the reductions in brain protein and DNA oxidation. Aging markedly lowered hepatic GR, SOD, CAT, and GPx activities, whereas NB23 increased all four enzymes, generally with larger responses in L3 and HT3 than in L1. Lactobacilli have been reported to enhance antioxidant defenses through strain- and model-dependent mechanisms; for example, L. plantarum AR501 increased NRF2-associated hepatic antioxidant enzyme expression in a D-galactose aging model [31]. Because NRF2 coordinates cellular responses to electrophilic and oxidative stress and its inducibility may decline with age [32], NRF2 is a reasonable candidate pathway. Nonetheless, the present study did not assess hepatic NRF2 abundance, nuclear translocation, phosphorylation, or canonical hepatic target-gene expression, and whole-brain *Nfe2l2* mRNA was not altered by treatment after multiplicity adjustment. It would therefore be premature to attribute the enzymatic effects to NRF2 activation. Changes in enzyme stability, cofactor availability, redox state, or post-translational regulation could also contribute and should be examined in follow-up experiments.

The femoral micro-CT results indicate partial preservation of trabecular organization rather than complete normalization of bone. Natural aging increased trabecular separation and pattern factor and lowered several indices of bone quantity and density. All NB23 groups had lower trabecular pattern factor than aged controls, L3 and HT3 had lower trabecular separation, and HT3 had higher BMD. In contrast, several other microarchitectural variables, including bone volume fraction and trabecular number, were not significantly restored relative to aged controls. Probiotic effects on bone have previously been linked to reduced inflammatory signaling and osteoclast activity. In ovariectomized mice, *L. paracasei*-containing preparations preserved cortical bone together with lower bone inflammatory cytokine expression, higher osteoprotegerin, and maintenance of bone-marrow regulatory T cells [33], while *Limosilactobacillus reuteri* reduced osteoclast-related signals and protected against ovariectomy-induced bone loss [34]. Treg/Th17 balance and the RANKL–RANK–OPG system have therefore been proposed as components of probiotic–bone crosstalk [35]. SCFAs can also suppress osteoclast metabolism and protect bone mass in mice [12]. These mechanisms are biologically plausible but were not measured here: circulating cytokines, marrow T-cell subsets, bone-turnover markers, RANKL/OPG, osteoblast function, and tissue-level SCFA exposure were not assessed. The bone findings, especially the HT3 BMD result, should also be viewed as preliminary because micro-CT analyses included only four mice per group.

The similar response pattern observed with HT3 and high-dose live NB23 is a preparation-level finding; it does not identify small molecules as the active agents or establish equivalence between L3 and HT3. This pattern is biologically plausible in light of strain-specific evidence: both live and heat-treated NB23 showed exercise-related activity in a prior mouse study [19], heat-killed *L. paracasei* D3-5 and an isolated cell-wall lipoteichoic-acid fraction showed activity in aging-related assays [14], and heat-killed TWK10 has been studied for exercise-related outcomes in humans [36]. These studies support the possibility that heat-treated preparations retain activity through different classes of constituents, but effects cannot be assumed to transfer across strains or preparations. The present study therefore interprets HT3 as a whole preparation associated with selected outcomes. Distinguishing cell-associated from soluble fractions, verifying residual viability, and identifying candidate active constituents will require preparation-specific characterization and targeted assays.

The microbiome, fecal-SCFA, and intestinal-transcript findings do not establish a gut-mediated explanation for the functional outcomes. None of the 325 taxon–outcome pairs survived FDR correction, and the analysis matrices do not permit assessment of whether nominal associations are independent of age or treatment group. *Cldn3* and *Cldn5* transcript levels are not functional measures of intestinal permeability; fecal SCFA concentrations do not establish systemic exposure; and intestinal Ppargc1a expression is not a direct measure of muscle mitochondrial function. The present findings do not support a causal microbiota–SCFA–intestinal barrier–host pathway. Although previous strain-specific studies provide a rationale for future investigation, direct assessment of intestinal permeability, systemic metabolite exposure, and tissue-level responses is needed to determine whether such mechanisms contribute to the effects of NB23. Prior strain-specific studies provide biological context for targeted investigation [14,19,37], but do not establish the same mechanism for NB23. Direct permeability, systemic metabolite, and tissue-level functional measurements would be needed to evaluate this hypothesis.

The intervention did not produce a consistent adverse clinical-chemistry pattern. Body weight remained stable among the aged groups, most serum biochemical measurements were similar among aged controls and NB23-treated mice, and the lower BUN concentrations in L3 and HT3 were not accompanied by changes suggesting impaired renal function. Representative H&E sections of the liver, heart, kidney, lung, epididymal adipose tissue, and brown adipose tissue did not reveal a consistent overt treatment-associated lesion. These observations are reassuring but do not constitute a comprehensive safety assessment. Histopathology was descriptive and lacked blinded semiquantitative scoring, and isolated changes in lung and epididymal fat-pad weights—particularly in HT3—require replication and mechanistic clarification. Accordingly, safety conclusions should remain limited to the measured 12-week outcomes and tested doses.

This study has several strengths, including the use of naturally aged rather than chemically induced aging, inclusion of a young reference group, parallel evaluation of live and heat-treated preparations, and integration of behavioral, biochemical, histological, skeletal, intestinal, and microbiome outcomes. Several limitations should also be emphasized. Only male mice were studied, limiting generalizability across sex. Female aging involves ovarian hormone transitions that can alter bone turnover, muscle physiology, immune signaling, oxidative stress, and microbial ecology; these sex-dependent processes were not represented here and warrant dedicated studies in female animals, including attention to reproductive stage. The endpoint structure was tiered: core behavioral and serum-biochemistry datasets included 10 mice/group, while focused specimen-specific assays included micro-CT (n = 4/group), brain and intestinal qPCR (n = 5/group, except two gene-specific contrasts with n = 4), fecal SCFAs (n = 5/group), and sequencing (n = 5/group). These smaller endpoints broaden tissue-level coverage but remain exploratory; per-pair correlation n could not be reconstructed from the matrix-level output. Whole-brain homogenization prevented region-specific interpretation. The Morris water maze assessment included acquisition latency and path length, which showed concordant treatment-associated patterns; however, no probe trial or swimming-speed outcome was collected, so retention and possible noncognitive influences cannot be fully evaluated [38]. Representative muscle and organ histology was not supported by blinded pathology scoring. Inflammatory cytokines, intestinal permeability, mitochondrial function, protein-synthesis and degradation pathways, bone-turnover markers, and cell-specific immune outcomes were not measured. Finally, the cross-sectional microbiome correlations cannot establish temporal direction or causality, and the study was not designed to demonstrate statistical equivalence between live and heat-treated NB23. The qPCR reanalysis was post hoc, and multiplicity was not controlled across the full collection of physiological endpoints. The body-weight analysis summarized change from baseline to final measurement rather than modeling the complete longitudinal profile. Correlation results may reflect between-group differences or compositional constraints. These limitations increase the need for independent replication.

## 5. Conclusions

Twelve weeks of supplementation with live or heat-treated *L. paracasei* NB23 was associated with improvements in selected functional and physiological outcomes in naturally aged male mice. Treatment groups showed shorter Morris water-maze acquisition latencies and path lengths and longer treadmill endurance; high-dose live and heat-treated preparations also increased grip strength. Changes in hepatic antioxidant activity and glycogen, whole-brain dopamine and oxidative-damage measures, gastrocnemius myofiber area, and selected femoral micro-CT indices provide supporting evidence, with the bone findings remaining exploratory because only four animals per group were assessed. Intestinal and brain gene-expression panels, fecal short-chain fatty acids, and FDR-adjusted host–microbiome correlations did not establish a gut-mediated mechanism. The similar response patterns of high-dose live and heat-treated NB23 do not demonstrate equivalence. No overt toxicity was detected in the measured outcomes under the tested doses and 12-week conditions, but this does not establish general safety. These findings support further, confirmatory investigation of NB23 rather than a comprehensive anti-aging claim; studies with both sexes, adequately powered primary outcomes, better-characterized preparations, and targeted mechanistic assays are needed to establish efficacy and translational relevance.

## Figures and Tables

**Table 1:** Body weight and daily food and water intake during 12 weeks of live or heat-treated *Lacticaseibacillus paracasei* NB23 supplementation in young and aged mice.

Parameter	YC	AC	L1	L3	HT3
Initial body weight (g)	24.3 ± 1.5	36.1 ± 2.4	36.3 ± 1.8	36.2 ± 2.1	35.8 ± 1.4
Week 1 body weight (g)	25.0 ± 1.4	36.0 ± 2.3	36.3 ± 1.6	36.3 ± 1.9	35.7 ± 1.5
Week 2 body weight (g)	25.7 ± 1.5	35.9 ± 2.3	36.0 ± 1.5	36.1 ± 2.0	35.6 ± 1.5
Week 3 body weight (g)	26.7 ± 1.7	36.2 ± 2.4	35.9 ± 1.6	36.0 ± 2.2	35.7 ± 1.8
Week 4 body weight (g)	27.5 ± 1.8	35.9 ± 2.4	36.0 ± 1.5	36.1 ± 2.3	35.5 ± 1.7
Week 5 body weight (g)	28.1 ± 1.8	36.2 ± 2.6	35.6 ± 1.5	36.0 ± 1.9	35.6 ± 1.6
Week 6 body weight (g)	27.2 ± 2.9	35.7 ± 2.5	35.4 ± 1.6	35.6 ± 2.0	35.4 ± 1.7
Week 7 body weight (g)	29.3 ± 1.7	36.1 ± 2.3	35.6 ± 1.5	36.0 ± 1.9	35.7 ± 1.5
Week 8 body weight (g)	30.1 ± 2.4	36.0 ± 2.4	35.7 ± 1.3	35.8 ± 1.9	36.1 ± 1.7
Week 9 body weight (g)	30.0 ± 1.7	36.2 ± 2.5	35.8 ± 1.4	35.8 ± 2.0	35.9 ± 1.4
Week 10 body weight (g)	30.3 ± 1.8	36.1 ± 2.3	35.8 ± 1.4	35.8 ± 2.4	35.7 ± 1.6
Week 11 body weight (g)	30.3 ± 1.7	35.9 ± 2.3	35.8 ± 1.4	35.8 ± 2.4	35.8 ± 1.7
Week 12 body weight (g)	30.4 ± 1.8	35.8 ± 2.3	35.8 ± 1.3	35.9 ± 1.9	36.3 ± 1.5
Final body weight (g)	30.6 ± 1.7	35.7 ± 2.4	36.4 ± 1.2	36.0 ± 1.8	36.2 ± 1.6
Water intake (mL/mouse/day)	6.3 ± 0.8	5.3 ± 0.4	5.3 ± 0.5	5.3 ± 0.7	5.4 ± 0.3
Food intake (g/mouse/day)	7.7 ± 2.1	5.2 ± 0.8	5.1 ± 0.4	5.2 ± 0.7	5.3 ± 0.5

Body-weight data are mean ± SD (n = 10 mice/group). Weekly values are descriptive; inferential comparisons were conducted on each mouse’s change from baseline to final measurement, using Welch’s *t*-test for YC versus AC and Dunnett-adjusted contrasts for L1, L3, and HT3 versus AC. Food and water intake are descriptive group-level observations and are not independent mouse replicates. YC, young control; AC, aged control; L1 and L3, low- and high-dose live NB23; HT3, high-dose heat-treated NB23.

**Table 2:** Effects of live and heat-treated *L. paracasei* NB23 supplementation on serum biochemical parameters in aged mice.

Parameter	YC	AC	L1	L3	HT3
AST (U/L)	64 ± 8	68 ± 15	66 ± 21	65 ± 13	67 ± 16
ALT (U/L)	41 ± 4 ^#^	47 ± 5 ^#^	45 ± 6	44 ± 4	45 ± 8
BUN (mg/dL)	21.9 ± 2.7 ^#^	28.7 ± 2.2 ^#^	27.1 ± 1.4	26.7 ± 1.8 *	25.2 ± 2.0 *
CREA (mg/dL)	0.43 ± 0.04	0.41 ± 0.03	0.41 ± 0.02	0.40 ± 0.02	0.41 ± 0.01
TP (g/dL)	5.6 ± 0.3	5.7 ± 0.4	5.6 ± 0.3	5.6 ± 0.4	5.6 ± 0.3
TC (mg/dL)	100 ± 15 ^#^	116 ± 15 ^#^	115 ± 9	113 ± 9	114 ± 9
TG (mg/dL)	94 ± 11	106 ± 20	104 ± 18	103 ± 14	102 ± 20
HDL-C (mg/dL)	73.4 ± 16.2	83.1 ± 9.4	81.0 ± 2.7	80.6 ± 5.1	80.3 ± 6.6
LDL-C (mg/dL)	16.9 ± 3.4 ^#^	22.0 ± 4.2 ^#^	19.2 ± 2.2	19.9 ± 2.0	19.6 ± 2.8
GLU (mg/dL)	154 ± 25	168 ± 15	169 ± 12	168 ± 14	169 ± 16

Data are presented as mean ± SD (n = 10 mice/group). YC was compared with AC using Welch’s two-sided *t*-test; the three aged treatment groups were compared with AC using Dunnett’s multiple-comparison test. Superscript ^#^ identifies the YC–AC age-reference contrast (*p* < 0.05); superscript * identifies a Dunnett-adjusted treatment-versus-AC contrast (*p* < 0.05). No all-pairs inference is implied. YC, young control; AC, aged control; L1 and L3, live NB23 administered at the 1× and 3× doses, respectively; HT3, heat-treated NB23 administered at the 3× dose. AST, aspartate aminotransferase; ALT, alanine aminotransferase; BUN, blood urea nitrogen; CREA, creatinine; TP, total protein; TC, total cholesterol; TG, triacylglycerol; HDL-C, high-density lipoprotein cholesterol; LDL-C, low-density lipoprotein cholesterol; GLU, glucose.

**Table 3:** Effects of live and heat-treated *L. paracasei* NB23 supplementation on femoral microarchitectural parameters in aged mice.

Parameter	YC	AC	L1	L3	HT3
TV (mm^3^)	3.65 ± 0.22	3.69 ± 0.18	3.67 ± 0.15	3.65 ± 0.22	3.66 ± 0.13
BV (mm^3^)	0.38 ± 0.07 ^b^	0.32 ± 0.02 ^a^	0.34 ± 0.02 ^ab^	0.34 ± 0.03 ^ab^	0.35 ± 0.04 ^ab^
BV/TV (%)	10.29 ± 1.69 ^b^	8.58 ± 0.56 ^a^	9.25 ± 0.88 ^ab^	9.45 ± 0.83 ^ab^	9.49 ± 1.20 ^ab^
BS (mm^2^)	22.21 ± 1.39 ^b^	17.24 ± 0.81 ^a^	18.42 ± 1.75 ^a^	18.79 ± 0.80 ^a^	18.72 ± 2.71 ^a^
BS/BV (mm^−1^)	60.83 ± 13.14	54.66 ± 4.72	54.68 ± 7.69	54.82 ± 4.77	54.41 ± 8.72
Tb.Th (mm)	0.065 ± 0.007	0.073 ± 0.006	0.069 ± 0.004	0.069 ± 0.003	0.073 ± 0.006
Tb.Sp (mm)	0.239 ± 0.031 ^a^	0.357 ± 0.019 ^c^	0.334 ± 0.030 ^bc^	0.308 ± 0.040 ^b^	0.290 ± 0.036 ^ab^
Tb.N (mm^−1^)	1.78 ± 0.53 ^b^	1.40 ± 0.03 ^a^	1.46 ± 0.14 ^ab^	1.60 ± 0.05 ^ab^	1.60 ± 0.17 ^ab^
Tb.Pf (mm^−1^)	16.11 ± 1.33 ^a^	24.96 ± 2.55 ^c^	21.23 ± 1.77 ^b^	19.48 ± 1.65 ^b^	19.19 ± 1.66 ^b^
SMI (index)	2.02 ± 0.09	2.02 ± 0.15	2.02 ± 0.06	2.03 ± 0.14	2.03 ± 0.07
BMD (g/cm^2^)	0.359 ± 0.046 ^c^	0.273 ± 0.017 ^a^	0.296 ± 0.032 ^ab^	0.296 ± 0.020 ^ab^	0.339 ± 0.027 ^bc^

Data are presented as mean ± SD (n = 4 mice/group). Statistical comparisons were performed using one-way ANOVA. Values within a row that do not share a superscript letter differ significantly (*p* < 0.05) according to the SAS letter-grouping output supplied with the dataset. YC, young control; AC, aged control; L1 and L3, live NB23 administered at the 1× and 3× doses, respectively; HT3, heat-treated NB23 administered at the 3× dose. TV, tissue volume; BV, bone volume; BV/TV, bone volume fraction; BS, bone surface; BS/BV, bone surface density; Tb.Th, trabecular thickness; Tb.Sp, trabecular separation; Tb.N, trabecular number; Tb.Pf, trabecular pattern factor; SMI, structure model index; BMD, bone mineral density.

## Data Availability

The original contributions presented in the study are included in the article/Supplementary Material, further inquiries can be directed to the corresponding author.

## Supplementary Materials

The following supporting information can be downloaded at: https://www.mdpi.com/article/10.3390/foods15203742/s1, Table S1: Organ weights; Figure S1: Organ histology; Figure S2: Fecal metabolite panels; Figure S3: Brain qPCR.
