# Supplementary material for: Live and Heat-Treated Lacticaseibacillus paracasei NB23 Improve Selected Functional and Physiological Outcomes in Naturally Aged Mice
Source: Foods. 2026 Oct 20;15(20):3742. doi: 10.3390/foods15203742 (PMC13704608; doi:10.3390/foods15203742)
Supplement: Supplementary file 1 [file foods-15-03742-s001.zip › Supplementary Figure S2.pdf]

Figure S2.

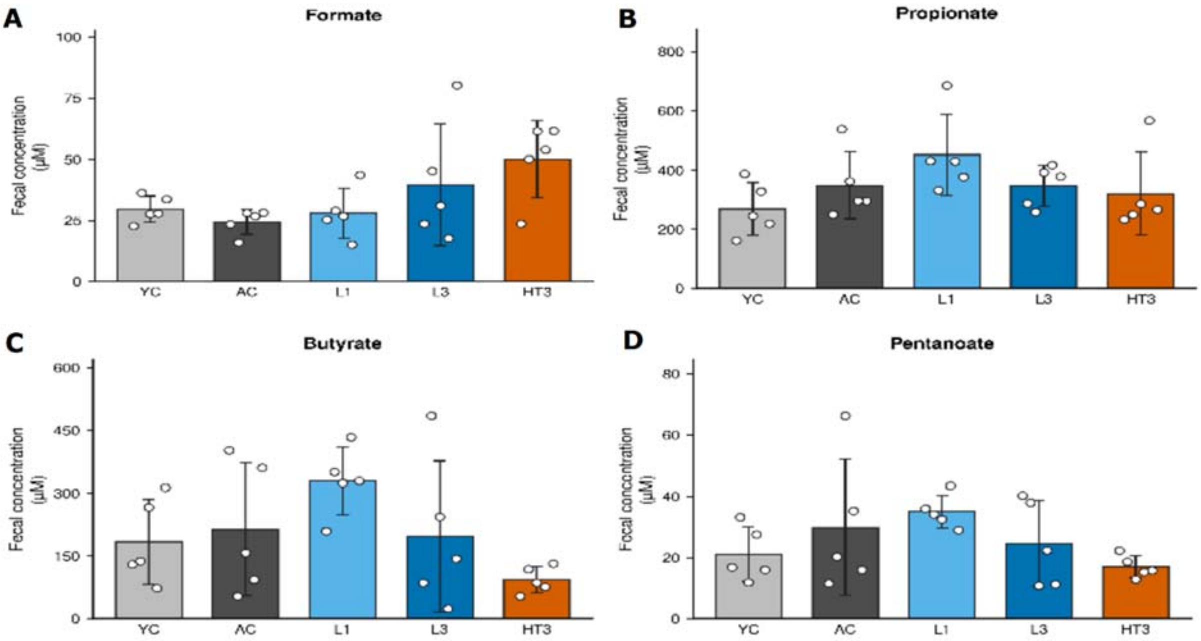

Figure S2. Fecal metabolites. Original fecal metabolite panels, retaining source labels **A–D**: formate, propionate, butyrate, and pentanoate, respectively. Panels are reproduced from the supplied main manuscript without reconstructing observations. Values are reported in µM as in the source. Bars show mean ± SD and points individual mice (n = 5/group). The original analysis reported no significant treatment differences after adjustment; individual assay records, extraction normalization, and the complete statistical output remain necessary for independent verification. YC, young control; AC, aged control; L1 and L3, low- and high-dose live NB23; HT3, high-dose heat-treated NB23.
