# Supplementary material for: Live and Heat-Treated Lacticaseibacillus paracasei NB23 Improve Selected Functional and Physiological Outcomes in Naturally Aged Mice
Source: Foods. 2026 Oct 20;15(20):3742. doi: 10.3390/foods15203742 (PMC13704608; doi:10.3390/foods15203742)
Supplement: Supplementary file 1 [file foods-15-03742-s001.zip › Supplementary Figure S3.pdf]

Figure S3.

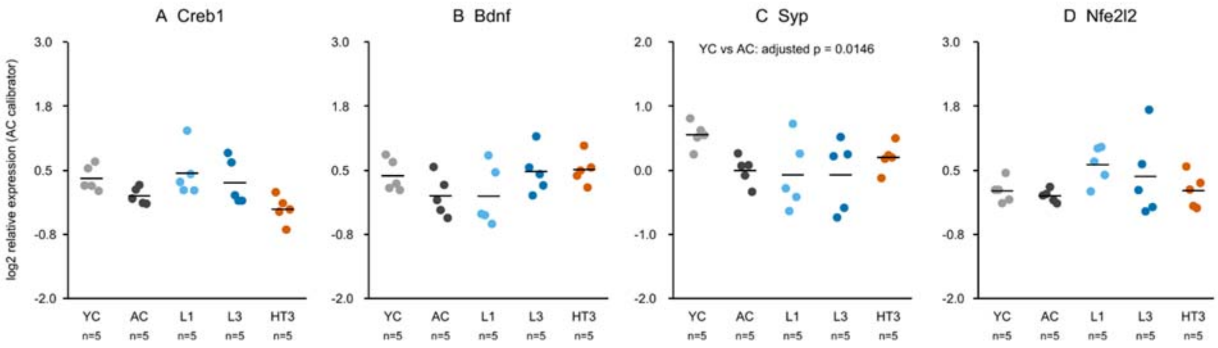

Figure S3. Brain transcripts. Exploratory whole-brain qPCR reanalysis for (A) Creb1, (B) Bdnf, (C) Syp, and (D) Nfe2l2. Each point represents one mouse (n = 5/group). ΔCt uses Actb as the reference gene and the AC mean as calibrator; plots show log2 relative expression. Horizontal lines indicate the mean. Two-sided Welch tests on ΔCt were adjusted with Benjamini–Hochberg correction separately across four age and twelve treatment contrasts. Syp differed between YC and AC (adjusted $p = 0.0146$). No treatment contrast met adjusted $p < 0.05$.
