# Supplementary material for: Live and Heat-Treated Lacticaseibacillus paracasei NB23 Improve Selected Functional and Physiological Outcomes in Naturally Aged Mice
Source: Foods. 2026 Oct 20;15(20):3742. doi: 10.3390/foods15203742 (PMC13704608; doi:10.3390/foods15203742)
Supplement: Supplementary file 1 [file foods-15-03742-s001.zip › Supplementary Table S1.pdf]

Table S1.

Table S1. Absolute and relative organ and tissue weights after 12 weeks of live or heat-treated Lacticaseibacillus paracasei NB23 supplementation in young and naturally aged mice.

Characteristic	YC	AC	L1	L3	HT3
**Liver (g)**	$1.35±0.12^{a}$	$1.73±0.12^{c}$	$1.67±0.09^{bc}$	$1.64±0.12^{bc}$	$1.62±0.09^{b}$
**Kidney (g)**	$0.37±0.04^{a}$	$0.43±0.05^{b}$	$0.43±0.03^{b}$	$0.43±0.03^{b}$	$0.44±0.03^{b}$
**Heart (g)**	$0.20±0.03^{a}$	$0.22±0.04^{ab}$	$0.25±0.06^{b}$	$0.22±0.03^{ab}$	$0.24±0.03^{b}$
**Lung (g)**	$0.25±0.04^{ab}$	$0.28±0.04^{b}$	$0.27±0.04^{ab}$	$0.28±0.04^{b}$	$0.24±0.02^{a}$
**GA**	$0.36±0.03^{b}$	$0.33±0.03^{a}$	$0.32±0.02^{a}$	$0.34±0.02^{ab}$	$0.35±0.03^{ab}$
**TA**	$0.12±0.02^{a}$	$0.14±0.03^{ab}$	$0.14±0.02^{b}$	$0.14±0.03^{a}$	$0.13±0.04^{a}$
**EFP (g)**	$0.06±0.02^{a}$	$0.07±0.02^{a}$	$0.08±0.01^{a}$	$0.07±0.02^{a}$	$0.09±0.02^{b}$
**BAT (g)**	$0.40±0.20^{a}$	$0.98±0.25^{b}$	$0.98±0.24^{b}$	$0.94±0.24^{b}$	$1.03±0.53^{b}$
**Brain(g)**	$0.43±0.03^{a}$	$0.44±0.03^{a}$	$0.44±0.03^{a}$	$0.44±0.02^{a}$	$0.45±0.03^{a}$
**Relative liver weight (%)**	$4.42±0.4^{a}$	$4.84±0.21^{b}$	$4.59±0.3^{ab}$	$4.56±0.18^{a}$	$4.49±0.39^{a}$
**Relative kidney weight (%)**	$1.20±0.10^{a}$	$1.21±0.13^{a}$	$1.17±0.10^{a}$	$1.19±0.06^{a}$	$1.21±0.12^{a}$
**Relative heart weight (%)**	$0.65±0.12^{a}$	$0.62±0.12^{a}$	$0.69±0.16^{a}$	$0.61±0.08^{a}$	$0.67±0.09^{a}$
**Relative lung weight (%)**	$0.83±0.12^{a}$	$0.79±0.13^{a}$	$0.74±0.1^{ab}$	$0.79±0.11^{a}$	$0.66±0.06^{b}$
**Relative GA weight (%)**	$1.19±0.10^{b}$	$0.92±0.08^{a}$	$0.89±0.08^{a}$	$0.94±0.06^{a}$	$0.96±0.11^{a}$
**Relative TA weight (%)**	$0.38±0.08^{a}$	$0.39±0.10^{a}$	$0.39±0.07^{a}$	$0.38±0.08^{a}$	$0.37±0.09^{a}$
**Relative EFP weight (%)**	$0.21±0.08^{a}$	$0.20±0.06^{a}$	$0.21±0.02^{ab}$	$0.19±0.05^{a}$	$0.26±0.06^{b}$
**Relative BAT weight (%)**	$1.29±0.63^{a}$	$2.73±0.63^{b}$	$2.67±0.63^{b}$	$2.59±0.57^{b}$	$2.83±1.45^{b}$
**Relative Brain weight (%)**	$1.40±0.11^{b}$	$1.24±0.14^{a}$	$1.2±0.08^{a}$	$1.23±0.11^{a}$	$1.24±0.11^{a}$

Data are presented as the mean ± SD (n = 10 mice/group). Statistical comparisons were performed using one-way ANOVA followed by Duncan's multiple-range test. Values within a row that do not share a superscript letter differ significantly ($p < 0.05$). Relative weight was calculated as tissue weight divided by terminal body weight × 100. YC, young control; AC, aged control; L1 and L3, aged mice receiving live NB23 at the 1× and 3× doses, respectively; HT3, aged mice receiving heat-treated NB23 at the 3× dose; GA, gastrocnemius muscle; TA, tibialis anterior muscle; EFP, epididymal fat pad; BAT, brown adipose tissue.
