# Supplementary material for: Live and Heat-Treated Lacticaseibacillus paracasei NB23 Improve Selected Functional and Physiological Outcomes in Naturally Aged Mice
Source: Foods. 2026 Oct 20;15(20):3742. doi: 10.3390/foods15203742 (PMC13704608; doi:10.3390/foods15203742)
Supplement: Supplementary file 1 [file foods-15-03742-s001.zip › Supplementary Figure S1.pdf]

Figure S1.

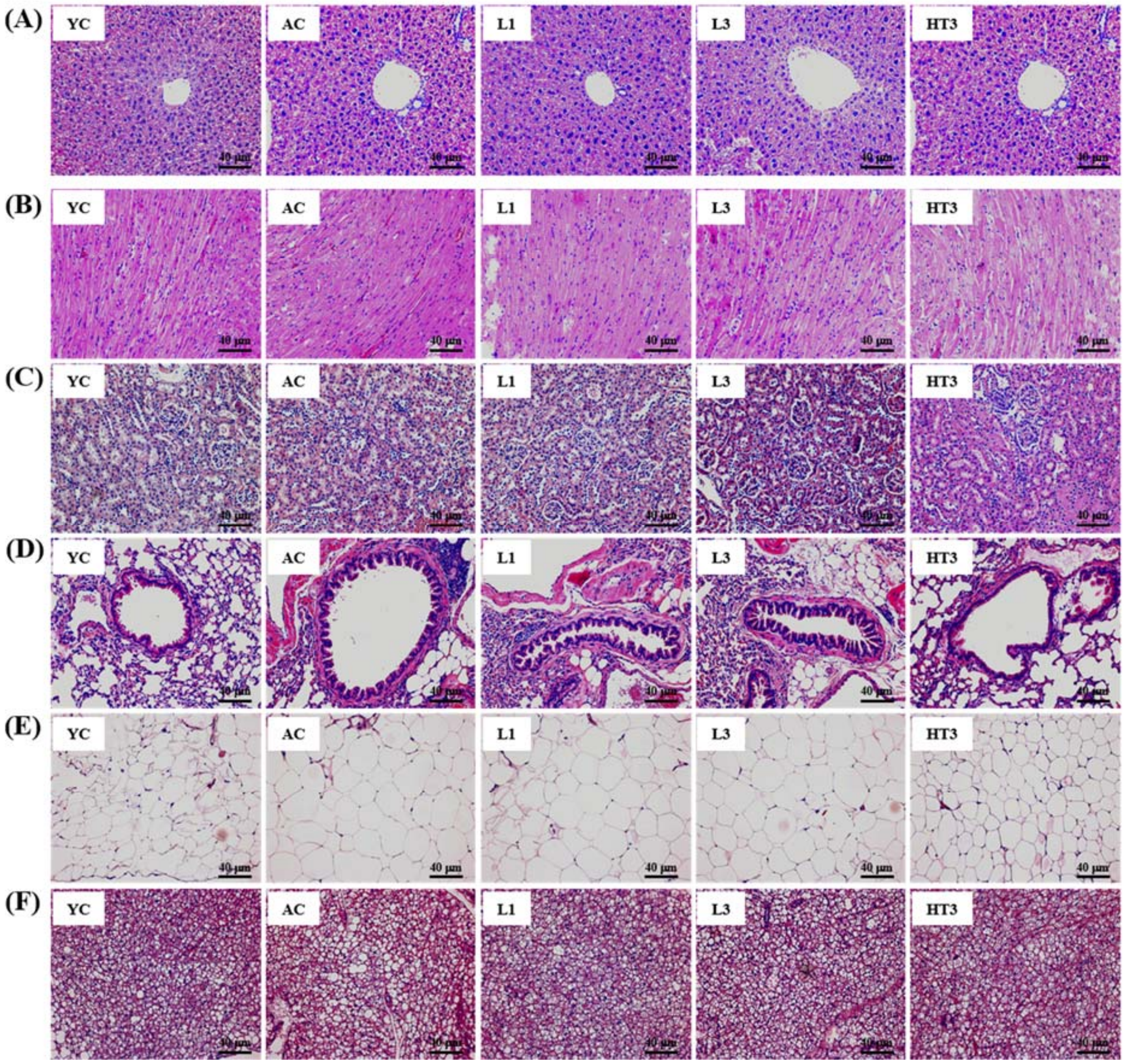

Figure S1. Organ histology. Representative histological appearance of major organs and adipose tissues after 12 weeks of live or heat-treated Lacticaseibacillus paracasei NB23 supplementation in young and naturally aged mice. Representative hematoxylin and eosin-stained sections are arranged from top to bottom as follows: (A) liver, (B) heart, (C) kidney, (D) lung, (E) epididymal fat pad, and (F) brown adipose tissue. Columns from left to right represent YC, AC, L1, L3, and HT3. Images are provided for descriptive histological assessment; no quantitative or semiquantitative histopathological scoring was performed. Scale bars = 40 μm. YC, young control; AC, aged control; L1 and L3, aged mice receiving live NB23 at the 1× and 3× doses, respectively; HT3, aged mice receiving heat-treated NB23 at the 3× dose; EFP, epididymal fat pad; BAT, brown adipose tissue.
